# Application of first-row transition metal complexes bearing 1,2,3-triazolylidene ligands in catalysis and beyond

**DOI:** 10.1039/d4cs00021h

**Published:** 2024-05-10

**Authors:** Wowa Stroek, Martin Albrecht

**Affiliations:** a Department of Chemistry, Biochemistry and Pharmaceutical Sciences, University of Bern Freiestrasse 3 CH-3012 Bern Switzerland martin.albrecht@unibe.ch

## Abstract

Triazole-derived N-heterocyclic carbenes, triazolylidenes (trz) have become an interesting alternative to the ubiquitous Arduengo-type imidazole-derived carbenes, in part because they are stronger donors, and in other parts due to their versatile synthesis through different types of click reactions. While the use of trz ligands has initially focused on their coordination to precious metals for catalytic applications, the recent past has seen a growing interest in their impact on first-row transition metals. Coordination of trz ligands to such 3d metals is more challenging due to the orbital mismatch between the carbene and the 3d metal center, which also affects the stability of such complexes. Here we summarize the strategies that have been employed so far to overcome these challenges and to prepare first-row transition metal complexes containing at least one trz ligand. Both properties and reactivities of these trz complexes are comprehensively compiled, with a focus on photophysical properties and, in particular, on the application of these complexes in homogeneous catalysis. The diversity of catalytic transformations entailed with these trz 3d metal complexes as well as the record-high performance in some of the reactions underpins the benefits imparted by trz ligands.

## Abstract 2

Triazole-derived N-heterocyclic carbenes, available *via* click reactions, are versatile ligands for first-row transition metals, leading to complexes with attractive photochemical properties and catalytic activity, some defining the state-of-the-art.

## Graphical abstract

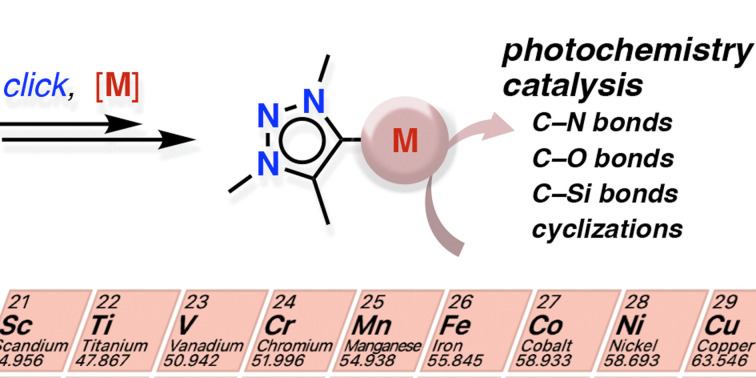

## Introduction

N-Heterocyclic carbene (NHC) complexes were reported for the first time in the 1960's by Wanzlick and Öfele.^1,2^ However, due to their low stability, NHCs remained by and large a laboratory curiosity until 1991, when Arduengo reported on stable and crystalline imidazole-2-ylidenes.^3^ This discovery facilitated NHCs to be used as ligands to virtually any metal and spurred their application in materials chemistry and in particular in catalysis.^4–14^ Subsequently, alternative strategies were discovered to stabilize NHCs and make them viable for diverse applications. Among those are cyclic (alkyl)(amino)carbenes (CAACs),^15,16^ (amino)(aryl)carbenes,^17^ cyclopropenylidenes^18^ and 1,2,3-triazolylidene (trz) systems.^19^ Although all these classes of carbene ligands have remained less popular than Arduengo-type imidazole-2-ylidenes, they are steadily gaining momentum.

**Scheme 1:**
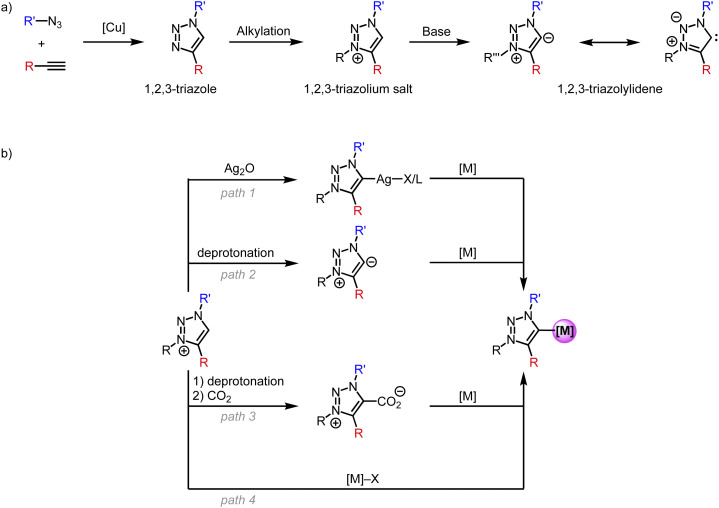
(a) General synthesis of 1,2,3-triazolylidene ligands; (b) strategies for the synthesis of trz transition metal complexes starting from the triazolium salt.

In particular, the triazole scaffold has become increasingly attractive in many domains, including organic chemistry, medicine, and biology, and therefore provides a versatile platform for carbene ligand synthesis.^20–28^ This overview focuses specifically on the use of triazole-derived carbenes as a subclass of NHCs for first-row transition metals. Following the first report on metal complexes featuring a trz ligand in 2008,^29^ this class of ligands has become more and more useful as a polyvalent support for transition metals and has disclosed original applications.^30,31^ This success is due, for a large part, to the simple synthesis of the triazole ligand scaffold through the copper-catalyzed azide–alkyne Huisgen cycloaddition (CuAAC) to form 1,4-disubstituted 1,2,3-triazoles (Scheme 1a).^24,26^ A less common 1,5-disubstitution is achieved when using alternative base- or ruthenium-catalyzed conditions.^32–34^ Subsequent alkylation of the N(3)-position affords the 1,2,3-triazolium salt, which features an acidic proton. Such triazolium salts are also accessible from clicking alkynes with a 1,3-diaza-2-azoniaallene salt.^35^ Deprotonation of the triazolium salt with a strong base affords the free trz ligand, which is usually unstable in its free form and has only been isolated when both nitrogens N(1) and N(3) are aryl-substituted.^19^ Due to their zwitterionic character they are often referred to as mesoionic carbenes (MICs), even though MICs are a much broader class than trz only and include, for example, also imidazole-4-ylidenes and 3-pyridylidenes. In addition, the carbene in trz systems is stabilized by only one neighboring heteroatom, whereas Arduengo-type NHCs benefit from the stabilization of adjacent nitrogens on both sides. This low heteroatom stabilization lowers the π-acceptor properties of trz systems even further.^36,37^ Simultaneously, the absence of the second adjacent heteroatom reduces the electron density redistribution through inductive effects and enhances the zwitterionic character, and these effects increase the carbanionic character of the carbenic carbon in trz ligands. This situation is reflected, for example, in the lower acidity of triazolium *vs.* imidazolium salts (p*K*_a_ ∼25 *vs.* 23), and in turn results in significantly stronger σ-donor properties of trz ligands compared to Arduengo-type 2-imidazolylidenes. Thus, the combination of less p-acceptor and more s-donor effects lead to a much stronger electron donor properties of trz compared to classic Arduengo NHCs, with obvious consequences, *e.g.* for promoting oxidations at the metal center.

Several methods have been developed for the metalation of triazolium salts to generate trz transition metals complexes (Scheme 1b). Transmetalation (path 1) is a popular method for 2nd and 3rd row transition metals and entails reaction of the triazolium salt with Ag_2_O to form a trz silver complex *ex* or *in situ* followed by addition of a transition metal precursor to give a trz transition metal complex.^29^ The silver complexes are often unstable.^38^ A complementary pathway relies on the formation of the free carbene (path 2) by deprotonation with a strong base, *e.g.*, KO*t*Bu or KHMDS (HMDS = hexamethyldisilazide, N(SiMe_3_)_2_^−^), followed by coordination to a transition metal center.^39^ Typically, the deprotonation is performed *in situ* as the free carbene is too unstable, but some examples show the isolation of a stable free carbene with subsequent coordination to a transition metal.^19,39^ Alternatively, the *in situ* formed free carbene is trapped with CO_2_ (path 3) to form an air-stable triazolium carboxylate.^40^ Upon addition of a transition metal, decarboxylation occurs to form the corresponding trz complex. Finally and most conveniently, direct metalation (path 4) is achieved by reacting the triazolium salt with a transition metal precursor containing an internal base, *e.g.*, [Ni(OAc)_2_], Cu_2_O, [Pd(OAc)_2_] or [Pd(acac)_2_].^41^

**Fig. 1:**
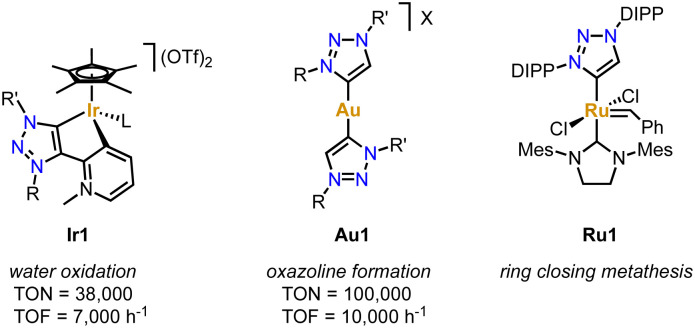
Examples of 2nd and 3rd row transition metal complexes bearing trz ligands that display extraordinary catalytic activity.

Due to their simple and essentially unlimited tuneability combined with their well-developed complexation methodologies, trz ligands have become increasingly popular over the last decade, in particular in combination with 2nd and 3rd row transition metals.^42–44^ Coordination of the trz to these metals forms a strong and largely covalent M–C_trz_ bond due to the soft character of both the metal and the carbene. Together with the pronounced σ-donor properties of the C_trz_ ligand, the coordinated transition metal is robustly coordinated and electron rich, which has been utilized in many catalytic transformations. For example, the trz iridium complexes Ir1 bearing a supporting pyridylidene and pyridine ligand, respectively (Fig. 1), are soluble in aqueous solution (a consequence of the zwitterionic ligand) and remarkably stable under acidic conditions. Even in 1 M HCl solution and elevated temperature, or upon addition of the strong oxidant (NH_4_)_2_[Ce(NO_3_)_6_] (CAN), the Ir–C_trz_ bond remains intact.^45^ Furthermore, the addition of CAN induces catalytic water oxidation, which involves the formation of higher valent Ir^IV^ or possibly even Ir^V^ intermediates. The accessibility of these high-valent species obviously benefits from the strong donor properties of the trz ligand. As a consequence, complex Ir1 reaches TONs up to 10 000 and TOFs around 300 h^−1^, which represented state-of-the-art performance at that time.^46^ The simple tunability of the trz ligands, allowed significant improvement of the water oxidation catalysis increasing the TON to 38 000^47^ and the TOF to 7000 h^−1^.^48^ Similarly, trz Ag complexes promoted unusual C–CN bond cleavage reactions,^49^ trz Au complexes such as Au1 provided excellent catalysts for C–C bond making for the construction of oxazolines,^50^ and trz Ru complex Ru1 offers a unique proton-triggered activation mechanism for the generation of an olefin metathesis catalyst that exceeds those of commercial systems (Fig. 1).^51^

**Fig. 2:**
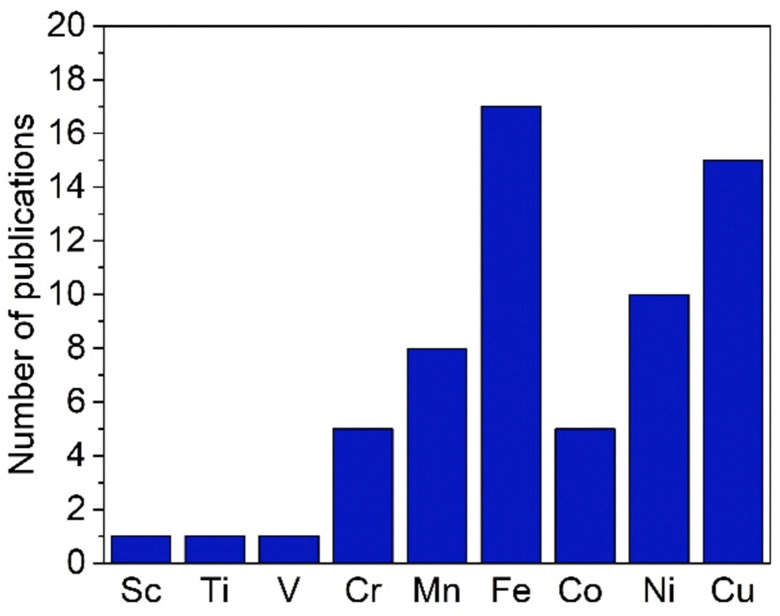
Total number of publications featuring a 1,2,3-triazolylidene complex with different first-row transition metals.

**Fig. 3:**
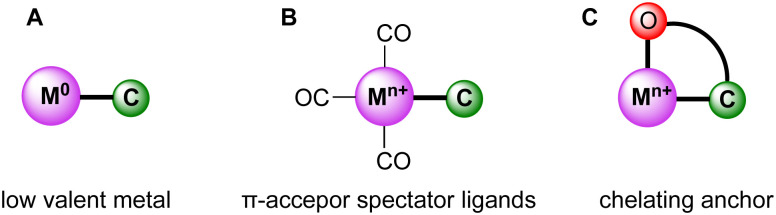
Schematic representation of strategies (A–C**)** to stabilize the bond between the trz ligand (labelled as C) and first-row transition metals (labelled as M).

While trz complexes with 2nd and 3rd row transition metals have been extensively studied, analogues with 1st row transition metals have been much rarer (Fig. 2),^30^ despite the beneficial attributes of trz ligands, *viz.* strong electron donation, simple steric and electronic tunability, bond stability, and increased water solubility. Combining these features with 1st row transition metals that are typically low cost and low toxicity, and highly abundant, offers attractive opportunities for the development of functional complexes for large-scale applications. However, the coordination chemistry of 1st row transition metals is far more challenging compared to their 2nd and 3rd row counterparts. While leveraging the strong electron donor properties as well as the steric and electronic tunability is intrinsic to the trz ligand and independent of the type of metal used, the bond stability with base metals changes drastically compared to the 4d and 5d analogues. A major complication arises from the bonding mismatch between the soft carbene and the metal center, which is typically classified as hard^52–57^ with first-row transition metals. The hard Lewis acidic character of 3d metals, especially the early ones, arises from their small atomic radius and typically high valency, which translates in predominantly ionic interactions.^58^ On the other hand, carbenes have a soft character, making them excellent ligands for covalent bonding. This hard–soft mismatch imparts only little stability, and additional strategies are often required for the successful synthesis of 3d carbene complexes (Fig. 3).

One strategy relies on the use of metal centers in low valence oxidation states, which reduces the hard character of the metal (A, Fig. 3). Similarly, the use of strongly π-accepting spectator ligands such as CO facilitate trz coordination by their complementary character with respect to the strongly σ-donating carbene ligands (B, Fig. 3). Moreover, π-accepting ligands withdraw electron density from the metal center and therefore stabilize low-valent oxidation states. As a third strategy, multidentate trz ligands that are functionalized with anchoring hard ionic ligands are beneficial as they provide the hard-hard interaction needed for robust coordination of first-row transition metals (C, Fig. 3). This chelation supports the trz coordination and lowers the substitutional lability of the complexes.

**Fig. 4:**
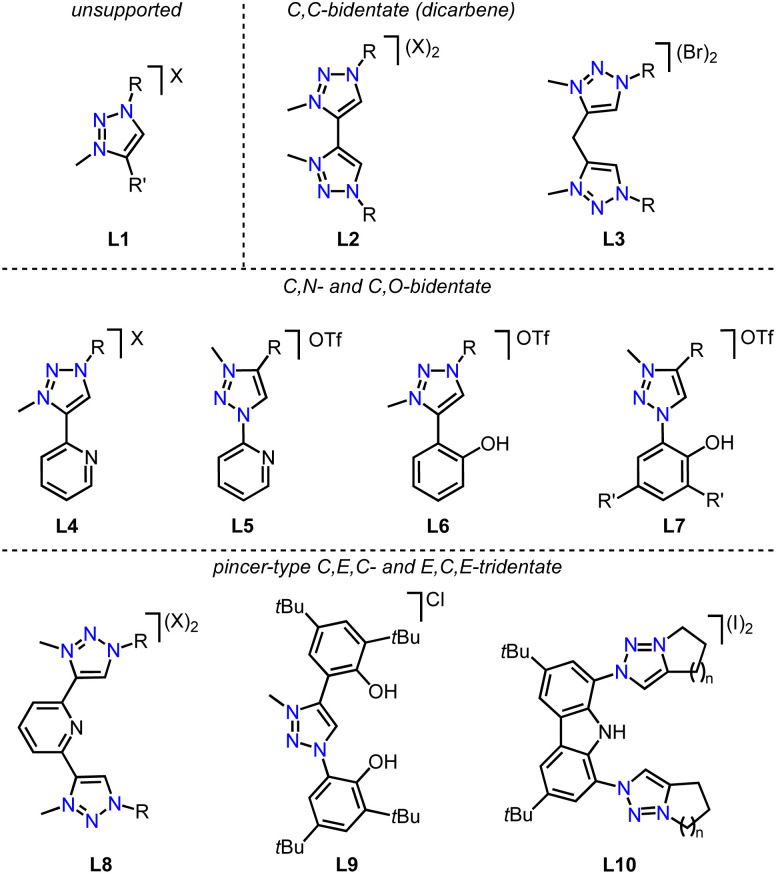
Recurring trz ligand motifs L1–L10 used for 3d transition metal complexation; R, R′ = alkyl, aryl; X = OTf, Br, I, BF_4_.

All three strategies have been successfully applied over the last few years to synthesize first-row transition metal complexes with trz ligands, which were often used then for catalytic or photochemical applications.^31^ Here, we summarize the progress in this area, following a 2018 review on trz complexes in general,^30^ and a review by Sarkar and coworkers covering the synthesis of several first-row transition metal trz complexes,^31^ yet with only scattered showcases of their applications. Furthermore, several themed reviews feature mainly imidazole-derived NHC complexes and also briefly mention trz analogues.^59–63^ Complementary to these reviews, we provide here a comprehensive overview focusing on trz complexes with first-row transition metals, with a particular emphasis on their properties, reactivity, and catalytic activity. An overview of the most recurring ligand motifs L1–L10 with the trz scaffold are displayed in Fig. 4.

## Early first-row transition metals

### Scandium

**Scheme 2:**
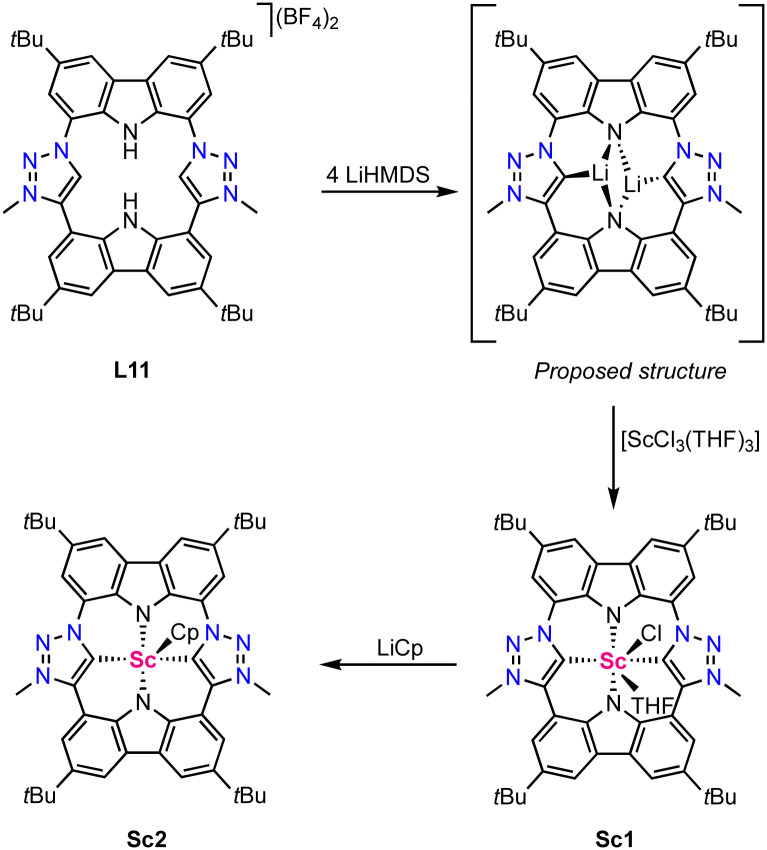
Synthesis of the scandium porphyrinoid complexes Sc1 and Sc2 featuring two trz ligands.

Only two scandium complexes bearing a mesoionic carbene ligand are known so far and have been reported by Kunz in 2021.^64^ The ligand precursor L11 features a porphyrinoid scaffold comprised of two mesoionic carbene moieties and two amide coordination sites (Scheme 2). Four-fold deprotonation with LiHMDS yields a di-lithium complex in which, according to a DFT optimized structure (DFT = density functional theory), each lithium coordinates to two amines and one carbene moiety. Transmetalation with [ScCl_3_(THF)_3_] (THF = tetrahydrofuran) affords the trigonal prismatic complex Sc1 with the ligand binding in a tetradentate fashion. The Sc–C_trz_ bond length is 2.26(1) Å according to crystallographic data. Notably, the scandium is positioned 0.99 Å above the porhpyrinoid plane, similar to related Sc(iii) porphyrine complexes.^65,66^ DFT-calculated Mulliken charges indicate that the dicarbene ligand derived from L11 is a significantly stronger electron-donor than tetraphenylporphyrin (TTP), as complex Sc1 has a lower positive charge than known [Sc(TPP)Cl] and also than the hypothetical complex [Sc(TPP)Cl(THF)].^66^ The coordinated THF and chloride of Sc1 are labile and have been successfully replaced by a cyclopentadiene (Cp) ligand in complex Sc2 upon reacting complex Sc1 with LiCp (Scheme 2).

### Titanium

**Scheme 3:**
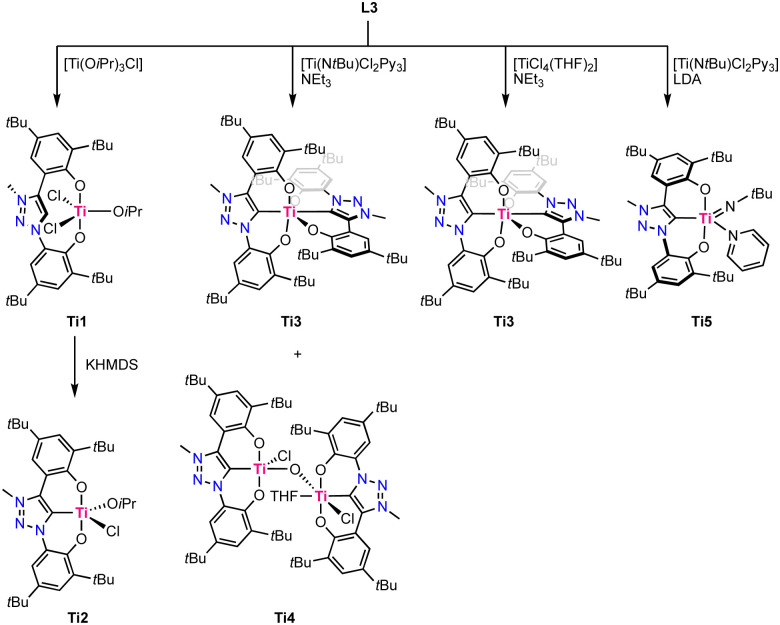
Synthesis of titanium trz complexes Ti1–Ti5.

In 2019, Hohloch reported the first titanium complexes containing a coordinated trz ligand.^67^ The used ligand precursor L9 is comprised of two phenol moieties and acts upon triple deprotonation as a *O*,*C*,*O*-tridentate chelate. Coordination of the ligand to titanium was performed using four different methods, resulting in different titanium complexes (Scheme 3). Direct metalation of ligand precursor L9 with [Ti(OiPr)_3_Cl] results in deprotonation of both phenol moieties and their subsequent coordination, forming Ti1. One isopropoxide remains coordinated to titanium and is not a strong enough base to deprotonate the triazolium site, which consequently remains uncoordinated, even at temperatures up to 110 °C. Upon treatment with KHMDS, deprotonation and coordination of the trz occurs to form complex Ti2. Using [Ti(N*t*Bu)Cl_2_Py_3_] as a precursor the in presence of NEt_3_ instead affords the octahedral homoleptic complex Ti3 where two ligands coordinate in a tridentate fashion to titanium. The homoleptic titanium complex Ti3 is also formed by using [TiCl_4_(THF)_2_] as a metal precursor in the presence of NEt_3_, though with this procedure also the bimetallic, oxo-bridging complex Ti4 is obtained as a side product. Its crystal structure reveals that one titanium adopts a square pyramidal geometry with a Ti–C_trz_ bond length of 2.111(5) Å, and the second titanium is octahedral due to coordination of THF and features a slightly longer Ti–C_trz_ bond of 2.130(5) Å. Using LDA (LDA = LiNiPr_2_) as a base instead of NEt_3_ results in the clean formation of the square pyramidal imido complex Ti5. Its crystal structure shows a severely distorted square pyramidal geometry (*τ*_5_ = 0.31) and again a Ti–C_trz_ bond length of 2.112(5) Å. Complex Ti3 is redox-active and displays two reversible oxidation processes at +0.18 V and +0.58 V (*vs.* Fc^+/0^, Fc = ferrocene). Both oxidations have been assigned to be ligand-centered, as titanium(iv) cannot be oxidized due to the absence of d-electrons whereas the two phenoxy moieties have the potential to be redox-active.^68^ Analogous behavior has previously been established with the imidazolylidene analogue of this complex.^69^

### Vanadium

**Scheme 4:**
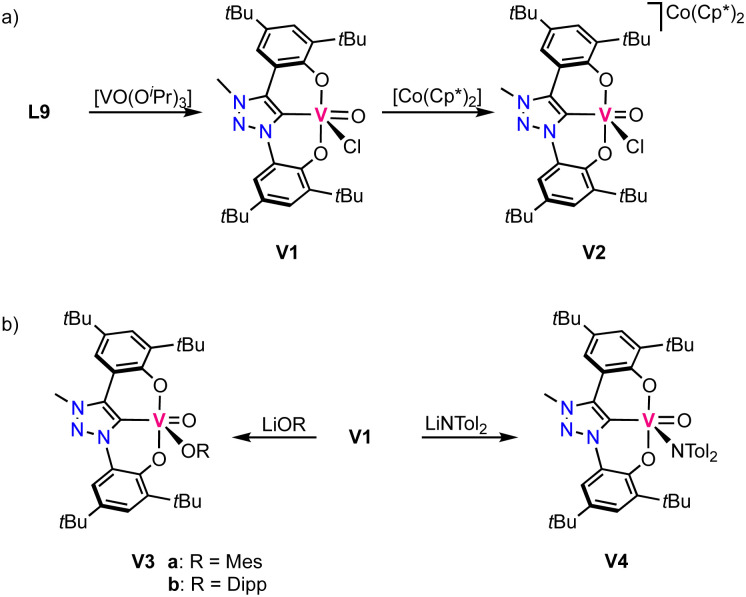
(a) Synthesis of vanadium(v) trz complex V1 and its reduced vanadium(iv) analogue V2; (b) reactivity of V1 in salt metathesis.

The first vanadium complexes with trz were reported by Hohloch and co-workers,^70^ using the same ligand precursor L9 previously reported for their titanium complexes.^67^ Contrary to titanium where the metal-bound isopropoxide is not sufficiently basic to deprotonate the triazolium salt, the isopropoxide vanadium precursor [VO(OiPr)_3_] induces deprotonation of both phenol groups and the triazolium unit to obtain complex V1 (Scheme 4a). The complex displays a square pyramidal geometry with the ligand coordinating in a tridentate fashion and a chloride and an oxo ligand occupying the remaining coordination sites. The V–C_trz_ bond length is 2.055(3) Å and considerably shorter than in the titanium analogues. Cyclic voltammetry of the complex shows two redox events: one oxidation at +1.05 V, assigned to be ligand-centered, and a metal-based reduction at −0.56 V (*vs.* Fc^+/0^). The latter has been confirmed by reducing V1 chemically with decamethylcobaltocene, which yields the vanadium(iv) complex V2 (Scheme 4a). EPR spectroscopy and DFT calculations reveal the presence of spin density at vanadium, in agreement with a vanadium-centered reduction to vanadium(iv). The crystal structure indicates no changes in the coordination environment after reduction apart from a slight elongation of the V–C_trz_ bond to 2.070(3) Å in agreement with higher electron density at the vanadium center. Complex V1 undergoes salt metathesis by chloride substitution in the presence of various lithium and potassium salts such as LiOMes (Mes = mesityl), LiODipp (Dipp = 2,6-Diisopropylphenyl) and LiN(tol)_2_, forming V3a,b and V4, respectively (tol = tolyl; Scheme 4b). However, reactions with LiNHMes, KPHMes, KSMes and anionic alkyl or aryl salts do not result in formation of any identifiable products.

**Scheme 5:**
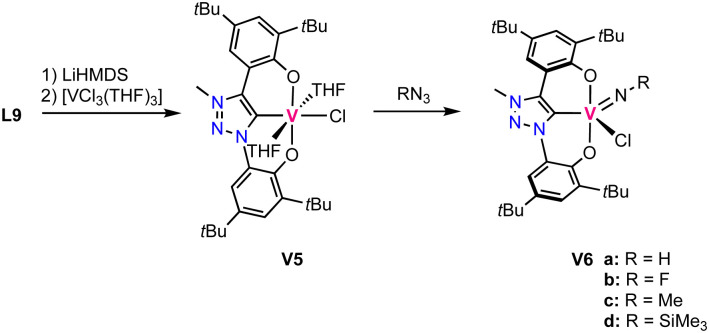
Synthesis of the low valent vanadium(iii) trz complex V5 and its oxidation with azides to form vanadium(v) imido complexes V6a–d.

The oxo ligand in complex V1 is tightly bound and does not exchange for an imido ligand upon reaction with isocyanates. Likewise, it is not transferred to various phosphines. Instead, a low valent vanadium(iii) complex is directly accessible by triple deprotonation of ligand precursor L9 with LiHMDS, followed by addition of [VCl_3_(THF)_3_] to afford the octahedral high-spin complex V5. This complex contains the ligand coordinated in a tridendate fashion and three other coordination sites occupied by two THF molecules and a chloride (Scheme 5). This complex is highly unstable and decomposes in the solid state even under inert atmosphere already at room temperature. At −40 °C the complex is, however, stable for several weeks. Reaction of this complex with aryl azides or TMS-N_3_ (TMS = SiMe_3_) yields the vanadium(v) imido complexes V6a–d (Scheme 5).

### Chromium

**Scheme 6:**
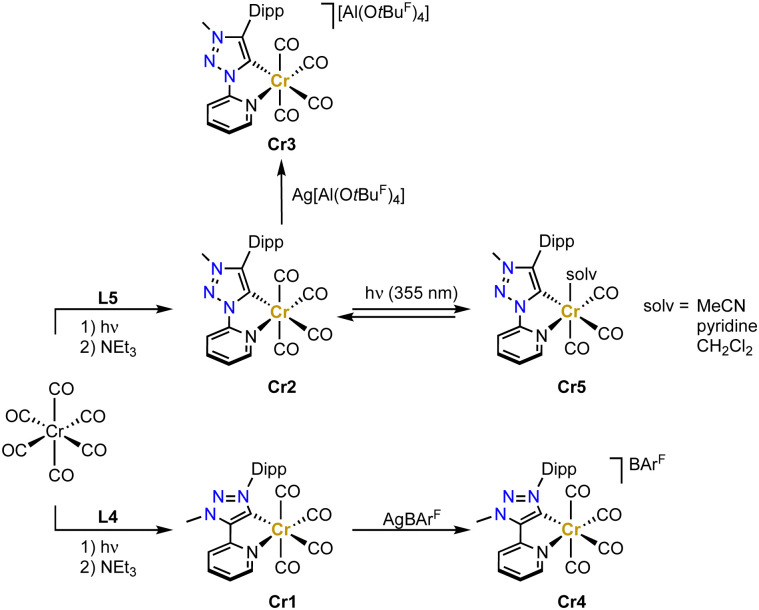
Synthesis of bidentate pyridyl-trz chromium(0) complexes Cr1 und Cr2 and their one electron oxidation complexes Cr3 and Cr4 as well as the proposed photoproduct Cr5 from time-resolved FTIR experiment (O*t*Bu^F^ = OC(CF_3_)_3_^−^; BAr^F^ = B(3,5-(CF_3_)_2_-C_6_H_3_)_4_^−^).

Chromium trz complex have not been reported until very recently.^71,72^ Complexes Cr1 and Cr2 have been synthesized by initially irradiating the [Cr(CO)_6_] precursor with UV-light in THF in order to form the solvento analogue, which is then treated with the Dipp-substituted ligand precursors L4 or L5 in the presence of NEt_3_ to afford the octahedral tetracarbonyl complexes containing the pyridyl-trz ligand coordinate in a bidentate fashion (Scheme 6). Crystallographic analysis indicates a slightly longer Cr–C_trz_ bond length in Cr2 than in Cr1 containing the C-bound pyridyl trz ligand (2.065(2) *vs.* 2.049(3) Å).^73^ Both complexes undergo a reversible oxidation around −0.2 V as well as a reversible and an irreversible reduction at about −2.2 and −2.8 V, respectively (potentials *vs.* Fc^+/0^). The reversible redox processes are slightly cathodically shifted for the C-bound pyridyl-trz complex compared to the N-bound analogue. EPR studies support an oxidation that is metal-centered, while the reversible reduction is centered on the trz ligand according to IR and EPR spectroelectrochemical analyses. Notably, this is the first time that a reduction on a triazolylidene was observed, similar behavior has subsequently also been detected with gold and selenium trz complexes.^74^ Chemical oxidation of Cr2 with AgPF_6_ induces gas release, while oxidation with Ag[Al(O*t*Bu^F^)_4_] (O*t*Bu^F^ = OC(CF_3_)_3_^−^) affords the chromium(i) complex Cr3 (Scheme 6). Analogous oxidation of Cr1 with AgBAr^F^ (BAr^F^ = tetrakis(3,5-bis(trifluoromethyl)-phenyl)borate) yields complex Cr4. In both complexes, the Cr–C(trz) bond distances remain unaltered when compared to their chromium(0) precursor complexes.

Upon excitation at 420 nm, complex Cr2 is photoactive and emits at *λ*_max_ ≈ 695 nm, with a tail into the NIR-II region.^75^ Furthermore, Cr2 undergoes photochemical CO dissociation and formation of the solvento complex Cr5 when irradiated at 355 nm according to time-resolved FTIR spectroscopy (Scheme 6). Since the dissociated CO remains in solution,^76^ the starting material is regenerated within 15 min in the dark state (in MeCN or CH_2_Cl_2_), yet slower (35 min) in pyridine due to its stronger coordination.

### Manganese

**Scheme 7:**
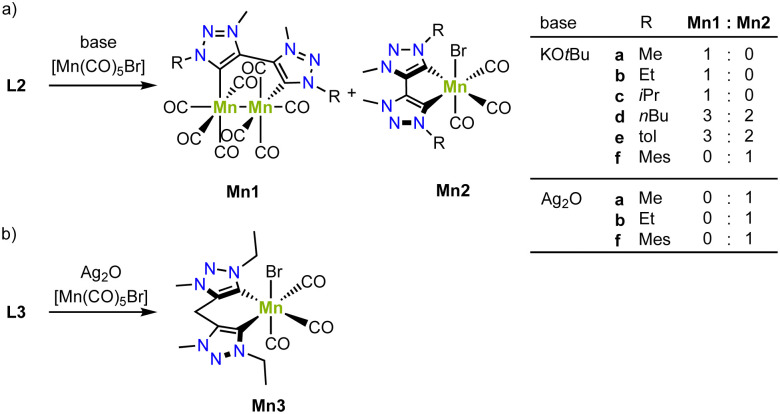
(a) Synthesis of bimetallic manganese(0) complexes Mn1 and monometallic manganese(i) complexes Mn2 bearing a bis trz ligand; (b) coordination of a bis trz ligand with a bridging methylene on manganes to form Mn3.

In collaborative work, the groups of Royo and us have developed a series of air- and moisture-stable mono- and bimetallic manganese complexes from the bis(trz) ligand precursors L2 (X = Br) with various substituents on the nitrogen.^77,78^ Deprotonation of the triazolium salt by KO*t*Bu in presence of [Mn(CO)_5_Br] affords either a monometallic manganese(i) complex, a bimetallic manganese(0) complex, or a mixture of the two, depending on the substituent on the nitrogen atom (Scheme 7a). For example, bis(trz) ligands with *n*Bu or *p*-tolyl substituents yield an approximate 2 : 3 mixture of mono- and bimetallic complexes (Mn2d/Mn1d, Mn2e/Mn1e). Only the mesityl substituent results in clean formation of the monometallic complex (Mn2f), while the Me, Et and iPr substituents afford exclusively the bimetallic complex (Mn1a–c). Interestingly, the formed bimetallic complex is paired with formation of bromine and reduction towards manganese(0). Contrary, when using a transmetalation route from silver for the formation of the manganese complex, the corresponding monometallic complex is cleanly formed (Mn2a, Mn2b, Mn2f). This method is also suitable for metalation of ligand precursor L3 (R = Et) with a methylene group between the two carbene units to form manganese complex Mn3 (Scheme 7b).^79^ These complexes showcase a combination of strategies A and B (*cf.*Fig. 3).

**Scheme 8:**
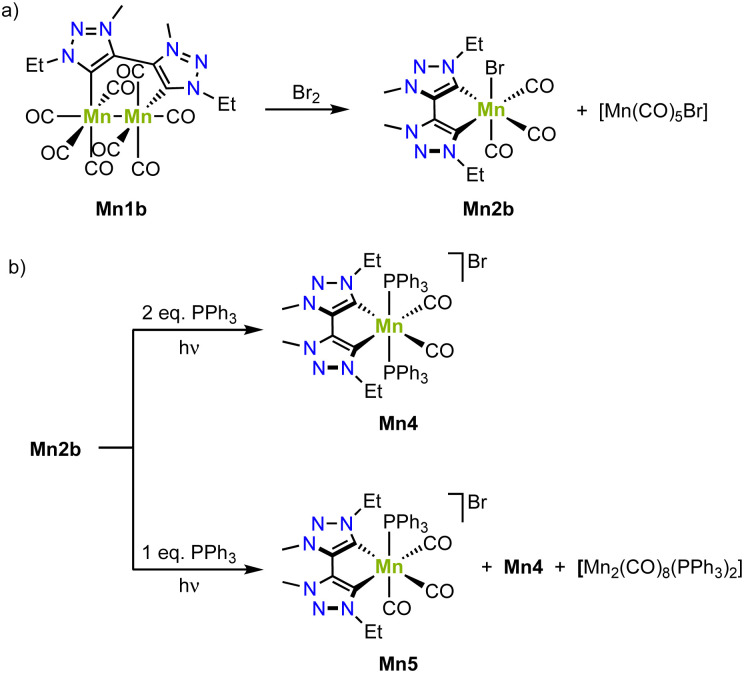
(a) Formation of the monometallic manganese(i) complex Mn2 by oxidation of the bimetallic manganese (0) complex Mn1; (b) photoreactivity of monometallic Mn2b in presence of PPh_3_ yields Mn4 or Mn5.

The bimetallic complex Mn1b is conveniently converted to the monometallic analogue Mn2b by reaction with bromine, which involves a one electron oxidation of each manganese(0) center to obtain one equivalent of monometallic complex Mn2b with an incorporated bromide and one equivalent of [Mn(CO)_5_Br] (Scheme 8a).^77^ Irradiation of complex Mn1b at 415 nm triggers loss of CO ligands and yields, in presence of two equivalents of PPh_3_, the cationic complex Mn4, where one axial carbonyl and the bromide ligands are replaced by two PPh_3_ (Scheme 8b). Using only one equivalent PPh_3_ affords Mn5 as the main product from a selective PPh_3_ for bromide ligand exchange. The redox chemistry of complex Mn1b demonstrates a reversible oxidation at +0.3 V and two irreversible reductions at −2.18 V and −2.45 V (*vs.* Fc^+/0^). FTIR spectroelectrochemistry reveals that even without applying any potential, the carbonyl stretching bands gradually change, which has been attributed to the substitution of a CO ligand with a solvent molecule.^80^ Based on FTIR spectroscopic monitoring and DFT calculations, both reductions are proposed to be metal-centered.

**Scheme 9:**
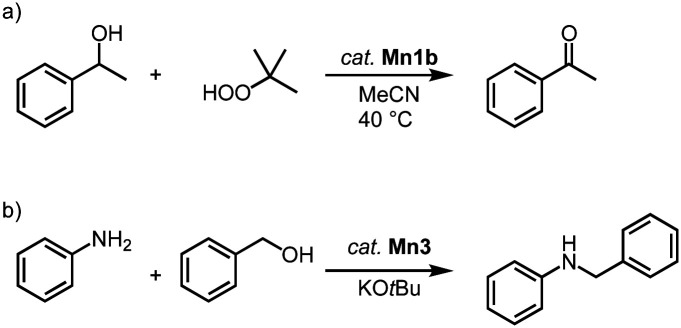
(a) Catalytic oxidation of 1-phenylethanol using TBHP, catalyzed by complex Mn1b; (b) catalytic *N*-alkylation of amines with alcohols using Mn3.^79^

Manganese trz complexes Mn2f and Mn1b catalyze the oxidation of alcohols in the presence of *tert*-butylhydroperoxide (TBHP) as sacrificial oxidant (Scheme 9a).^78^ Thus, 1-phenylethanol is fully converted to acetophenone within 2 h by catalytic amounts of Mn1b (1 mol%) at 40 °C with an initial turn over frequency TOF_ini_ = 72 h^−1^. The catalytic activity of the monometallic complex Mn2f is significantly lower (TOF_ini_ < 24 h^−1^, only 22% conversion after 2 h). Complex Mn1b catalyzes the oxidation of a variety of secondary alcohols including linear and cyclic aliphatic alcohols in 72–99% yield. Benzyl alcohol is oxidized predominantly to benzaldehyde (84%), with only 7% overoxidation to benzoic acid. However, primary aliphatic alcohols are not converted under these conditions.

In addition, complexes Mn1b and Mn3 are active in the catalytic *N*-alkylation of amines with alcohols (Scheme 9b).^79^ Thus, aniline alkylation with benzyl alcohol at 50 °C in the presence of KO*t*Bu and 1.5 mol% catalyst loading affords 42% *N*-benzylaniline within 24 h when complex Mn3 is used, yet only 15% yield are obtained with complex Mn1b. Increasing the temperature to 100 °C increases the yields to 99% and 45%, respectively. Lowering the amount of base from 1 to 0.5 equivalent results in a 99% yield with Mn3 after just 2 h, while the absence of the methylene bridge in the catalyst reduces the yield to 22%. Various benzylic and aliphatic alcohols are successfully converted, though replacing aniline with aliphatic amines gives only very low yields.

**Scheme 10:**
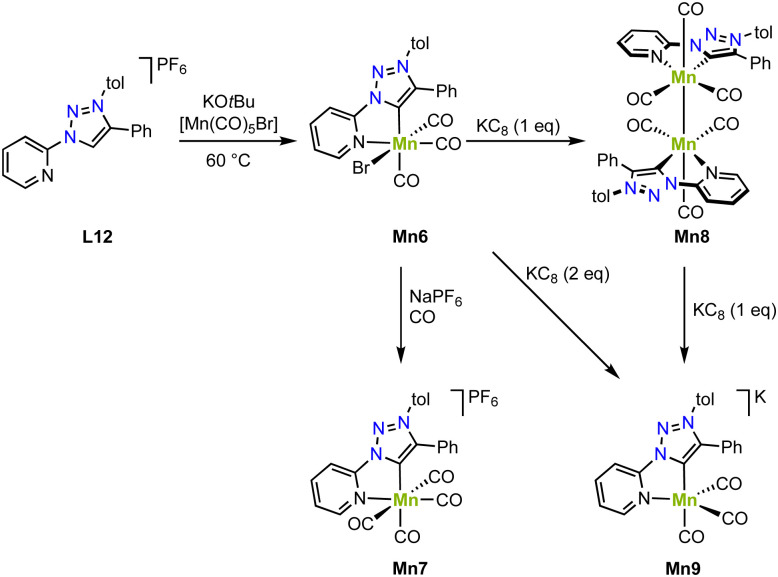
Synthesis of manganese complexes Mn6–9 with a *C*,*N*-chelating trz complex and in formal oxidation states of +1, 0, and −1.

Piers and coworkers have shown that deprotonation of the ligand precursor L12 with KO*t*Bu and *in situ* reaction with [Mn(CO)_5_Br] yields the diamagnetic manganese complex Mn6 containing a *C*,*N*-chelating trz ligand (Scheme 10).^81^ The crystal structure shows an octahedral geometry with a Mn–C_trz_ distance of 1.9958(18) Å. Reaction of this complex with NaPF_6_ under a CO atmosphere induces bromide substitution by a CO ligand and affords Mn7 with a slightly longer Mn–C_trz_ bond of 2.021(5) Å. One electron reduction of Mn6 with KC_8_ gives the new diamagnetic manganese(0) complex Mn8, which is dimeric and features a shorter Mn–C_trz_ bond (1.984(1) Å; Scheme 10). A Mn–Mn bond is indicated by the 3.0939(5) Å distance of the manganese centers and is consistent with the diamagnetic nature of Mn8. Interestingly, the complex also shows an EPR signal at *g* = 2.038, revealing one unpaired electron at a Mn(0) center. This behavior has been attributed to an equilibrium between the dimer and monomer in solution. Moreover, reduction of Mn6 with two rather than one equivalent KC_8_ yields the anionic and monomeric manganese complex Mn9 (Scheme 10). The crystal structure unveils an even shorter Mn–C_trz_ bond length of 1.9681(16) Å, in line with further reduction of the Mn center to manganese(−1). The crystal structure also indicates significant loss in aromaticity in the pyridyl ring, consistent with redox non-innocent behavior of the ligand. Also, the DFT calculated orbitals show significant ligand contribution towards the HOMO. The exact electronic structure may thus be comprised of either a Mn(i) center with a doubly reduced ligand, a Mn(−i) center with an unmodified ligand, or a Mn(0) center with a partially reduced ligand, with both electrons antiferromagnetically coupled to give in an open shell singlet structure. In agreement with these chemical redox reactivities, Mn6 displays two reduction processes in cyclic voltammetry at −1.57 and −1.87 V (*vs.* Fc^+/0^), assigned to formation of Mn8 and Mn9, respectively. In the presence of 2,2,2-trifluoroethanol and CO_2_, a significant increase in current of the second reduction wave is observed, indicative for electrocatalytic CO_2_ reduction. Electrolysis for 3.5 h at −1.54 and −1.94 V (*vs.* Fc^+/0^), yields, after a 30 min activation, CO in faradaic efficiency of 89 ± 10% and 90 ± 13%, respectively. The maximum TOF increases with overpotential and is 6.5 s^−1^ and 170 s^−1^, respectively. The high TOF at lower potential impacts catalyst stability, however, and after 24 h, the faradaic efficiency is reduced to 20 ± 9%, whereas at −1.54 V, the faradaic efficiency remains essentially quantitative.

**Scheme 11:**
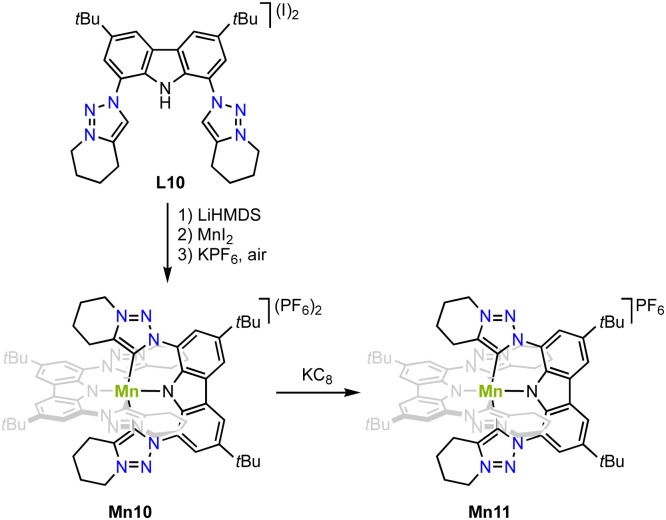
Synthesis of the octahedral manganese(iv) complex Mn10 with two pincer ligands that contain trz “arms” and its one electron reduction complex Mn11.

Strategy C (*cf*Fig. 3) is successful for stabilizing higher valent and air-stable manganese(iv) complex Mn10. Metalation of the carbazole-derived pincer ligand featuring two trz ‘arms’ is achieved upon deprotonation of pincer ligand precursor L10 with LiHMDS followed by *in situ* addition of MnI_2_ (Scheme 11).^82^ Crystallographic analysis indicates an octahedral complex and Evans' method confirms the presence of three unpaired electrons as expected for octahedral Mn^4+^ complexes. The Mn–C_trz_ bond length is 2.08(1) Å. Complex Mn10 is electrochemically very versatile and displays four different redox events and hence five possible oxidation states with reductions at −0.55 and −2.02 V, and oxidations +0.78 V and +1.30 V (*vs.* Fc^+/0^). So far only the first reduction product has been chemically accessible upon treating Mn10 with KC_8_. The Mn–C_trz_ bond lengths remain unaltered upon reduction to complex Mn11. EPR spectroelectrochemical analyses and DFT and CASSCF calculations suggest that the two reductions are metal-centered, while both oxidations occur on the ligand.

**Scheme 12:**
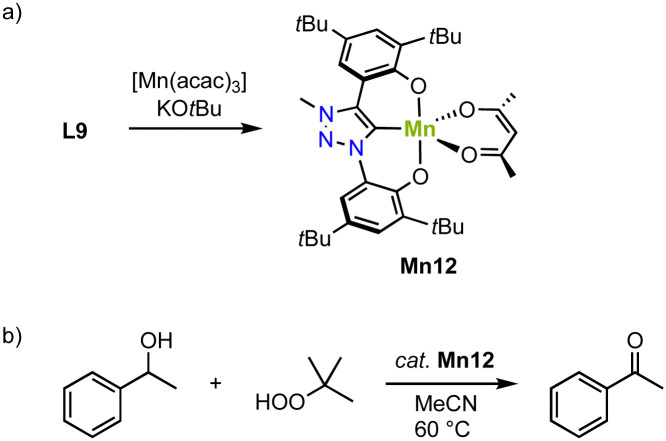
(a) Synthesis of the Mn(iii) *O*,*C*,*O*-pincer trz complex Mn12; (b) catalytic oxidation of 1-phenylethanol using TBHP, catalyzed by Mn12.

An alternative approach to stabilize higher-valent Mn in trz complexes uses the OCO pincer motive. Thus, reacting ligand precursor L9 with [Mn(acac)_3_] and KO*t*Bu, results in the formation of the paramagnetic complex Mn12 (Scheme 12a).^83^ The crystal structure reveals coordination of the tridentate pincer ligand and a bidentate acac ligand. The square pyramidal geometry is significantly more distorted (*τ*_5_ = 0.47) compared to the analogue featuring an imidazolylidene donor (*τ*_5_ = 0.18). The Mn–C_trz_ bond length, 1.9550(14) Å, is the shortest Mn–C_trz_ bond length known so far.

Complex Mn12 catalyzes alcohol oxidation with TBHP as terminal oxidant. For example, reaction of 1-phenylethanol with 1 mol% Mn12 at 80 °C results in high initial rates (TOF_max_ = 540 h^−1^) and an 80% yield to the corresponding ketone after 8 h (Scheme 12b). The complex is slightly more active than the imidazolium analogue, which under identical conditions reaches TOF_max_ = 500 h^−1^ and a lower 69% yield. This activity is about seven times higher than low-valent Mn trz complex Mn2b.^78^ Lowering the temperature to 60 °C reduces the initial reaction rate (TOF = 290 h^−1^), yet increases the overall yield to 94%. Rate analyses suggest gradual catalyst decomposition, which is accelerated at higher temperatures. At 0.1 mol% of catalyst the maximum turnover number (TON) is 400. Various functional groups in *para*-position of phenylethanol as well as secondary aliphatic alcohols are oxidized by Mn12, albeit some at lower yields (32–57%). Mechanistic studies using IR-spectroscopy, isotope labeling (KIE), and Hammett correlations suggest the formation of a Mn(v) oxo species upon oxidation with *t*BuOOH and rate-limited hydrogen atom abstraction (HAA) from the metal-bound alkoxide to form the ketone product.

## Late first-row transition metals

### Iron

**Scheme 13:**
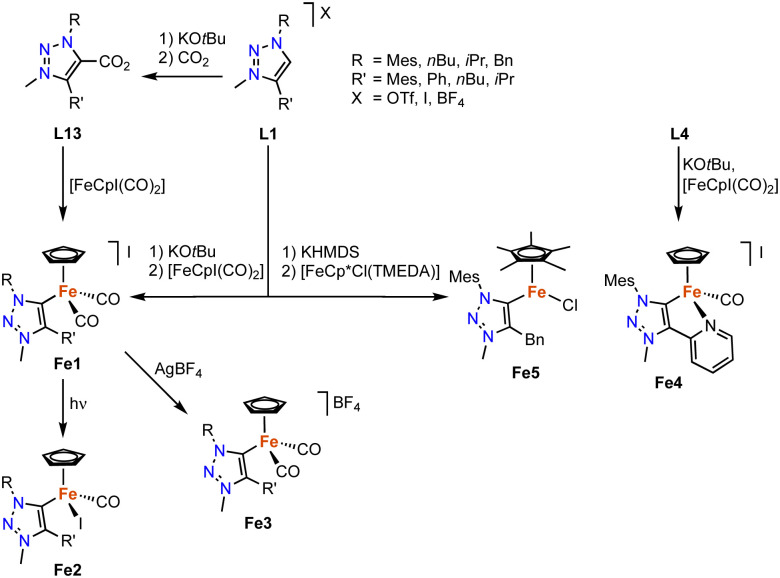
Synthesis of various iron piano-stool trz complexes Fe1–5*via* base-mediated metalation and decarboxylation.

Iron-based trz complexes have initially been focused on piano-stool systems. The first piano-stool iron complexes containing mono- and bidentate trz ligands with various wingtip groups on the carbon and nitrogen atom have been synthesized by deprotonation of the triazolium salt L1 (various R, R′ and X) with KO*t*Bu, followed by addition of the [FeCpI(CO)_2_] precursor to obtain cationic complexes Fe1 (Scheme 13).^84^ An alternative method involves the reaction of the deprotonated ligand with CO_2_, to yield triazolium carboxylate L13 (R = Mes, R′ = *n*Bu) as an air- and moisture-stable carbene precursor,^85^ which upon treatment with [FeCpI(CO)_2_] also yields the di(carbonyl) complex Fe1.^40^ This complex is not stable and slowly transforms to the neutral complex Fe2, a step that is accelerated by irradiation with UV light. Alternatively, the di(carbonyl) complex is stabilized by counterion exchange, for example with AgBF_4_ to yield complex Fe3. Complex Fe4 containing a bidentate trz-pyr ligand is obtained using the same deprotonation procedure as for Fe1, using L4 instead (R = Mes, X = I), and subsequent iodide abstraction with AgBF_4_.

**Scheme 14:**
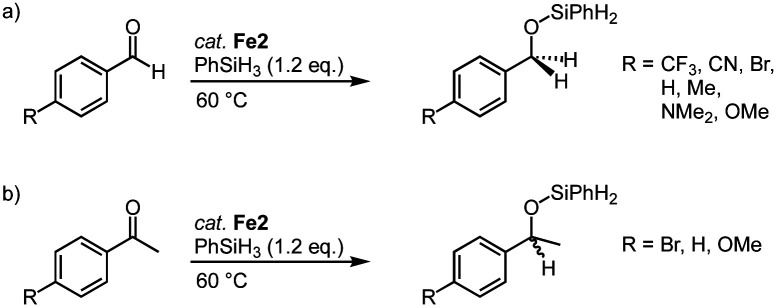
Trz iron complexes for the hydrosilylation of carbonyl functional groups including (a) *para*-substituted benzaldehydes; (b) *para*-substituted acetophenones.

Complexes Fe2, with trz ligands containing various wingtip groups, are active in the catalytic hydrosilylation of benzaldehyde with phenylsilane at 1 mol% catalyst loading (Scheme 14a).^40,84^ At 60 °C, the complexes reach conversions above 90% in 1 h. The different wingtip groups on the trz have only a minimal effect on the catalytic efficiency. The catalytic profile features an induction period in the first 30 minutes, after which the rate of the catalysis increases drastically to a TOF_max_ = 200 h^−1^. Interestingly, the bidentate trz ligand in complex Fe4 is significantly less active and reaches only 17% conversion under identical conditions. The functional group tolerance of complex Fe2 (R = Mes; R′ = *n*Bu) includes, among others, nitriles, amines, and ethers (Scheme 14a). Reaction progress analysis indicates that both initiation periods and catalytic rates depend on the functional group, though consistently full conversion is reached within 1 h. The rates are not correlated to classic Hammett parameters. Bromo-substituted benzaldehyde is the most active substrate in the series and is fully converted within 15 min even at 0.1 mol% catalyst loading, resulting in a TOF_max_ = 14 400 h^−1^. Also, ketones such as acetophenones are hydrosilylated (Scheme 14b), however, longer reaction times are required to achieve high conversion. In agreement with the tendency of first-row transition metals to often undergo single electron processes,^68^ control reactions with TEMPO or BHT as additive markedly decrease the hydrosilylation of bromo-benzaldehyde, (9% and 15% conversion, respectively, compared to full conversion without any additive; TEMPO = 2,2,6,6-tetramethylpiperidine-N-oxide, BHT = butylated hydroxytoluene). Moreover, stoichiometric experiments strongly suggest that catalyst activation involves CO dissociation and carbonyl substrate coordination.^40^

**Scheme 15:**
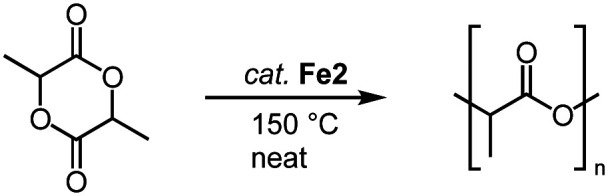
Polymerization of LA to PLA using iron piano-stool complex Fe2.

This catalyst activation mode also enables the conversion of other substrates containing C=O bonds. For example, complexes Fe2 also catalyze the polymerization of lactide (LA) to polylactic acid (PLA; Scheme 15).^86^ Even low catalyst loading (0.02 mol%; R = Mes; R′ = *n*Bu) convert neat *rac*-LA within 2 h at 150 °C to 82% PLA. The resulting polymer is characterized by a number-average molecular weight that is about half of that expected from living polymerization (*M*_n_ = 48 kg mol^−1^) and a polydispersity of 1.9, attributed to post-polymerization transformations. Of note, Fe2 displays a rate of polymerization twice as high as the most active iron catalyst known to date.^87^ Also the activity of the catalyst is almost an order of magnitude higher than the industrially used Sn(Oct)_2_. Changing both wingtip groups to iPr groups decreases the polymerization activity and affords only 58% conversion. Technical grade LA is compatible with complex Fe2 for polymerization, indicating robustness of the catalyst towards impurities of water and lactic acid. Interestingly, polymerization of recrystallized LA lowers the conversion from 91% to 74% (0.1 mol% catalyst, 2 h), though activity is fully restored when BnOH is added to the recrystallized LA. These observations are in agreement with an active role of ROH in the initiation of polymerization. Kinetic studies indicate a first-order rate dependence in catalyst concentration and point to a coordination insertion mechanism. Such a mechanism is also supported by MALDI-ToF MS measurements of the polymers (MALDI-ToF = matrix-assisted laser desorption ionization−time-of-flight), which show an iron-trz fragment as end group.

**Scheme 16:**
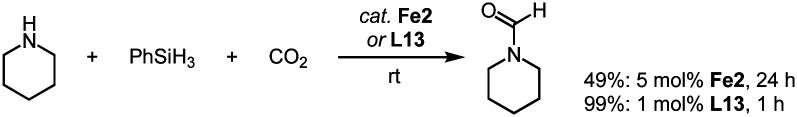
Reduction of CO_2_*via* hydrosilylation in the presence of piperidine catalyzed by complex Fe2 and by the free carbene derived from L13.

The reactivity of complexes Fe2 towards C=O units also allows CO_2_ to be reduced through hydrosilylation. Specifically, reaction of piperidine with PhSiH_3_ under a CO_2_ atmosphere produces the corresponding formamide already at room temperature (Scheme 16).^88^ Ligand variation indicates that the trz with Mes and *n*Bu wingtip groups imparts the highest catalytic activity and reaches 49% yield after 24 h at 5 mol% loading of Fe2. Optimization of the reaction conditions, especially the batchwise addition of the silane improves the conversion and leads to quantitative formylation of the amine. Remarkably, the metal-free trz-CO_2_ adduct L13 is considerably more active than the iron complex Fe2 and accomplishes full conversion after 12 h even at moderate 1 mol% loading. The high activity of the free trz might suggest that complex Fe2 only acts as a source for carbene release. However, mechanistic studies demonstrate that the Fe–C_trz_ bond is robust under catalytic conditions and that the hydrosilylation occurs at the iron center.

**Scheme 17:**
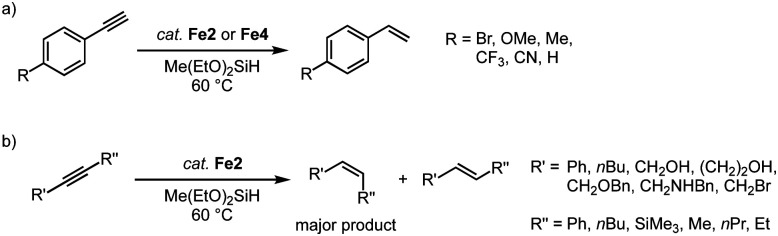
Selective semi-hydrogenation of alkynes catalyzed by complexes Fe2 and Fe4: (a) *para*-substituted phenylacetylene substrates yield substituted styrenes; (b) internal alkynes yield olefins with high *Z*-selectivity.

The hydrosilylation activity of these piano-stool complexes has been extended to the catalytic semi-hydrogenation of alkynes using silanes.^89^ Reaction of phenylacetylene with methyldiethoxysilane with 7 mol% Fe2 affords the semi-hydrogenated styrene in high yields (Scheme 17a). Smaller wingtip groups (R = Mes, R′ = *n*Bu) on the trz ligand of Fe2 induce a slightly higher initial activity compared to larger wingtip group (R = Mes, R′ = Mes/Ph). However, at extended reaction times, the larger wingtips groups impart higher robustness and result in full conversion after 24 h, while the smaller wingtip groups only reached 93%. Again, the bidentate trz in complex Fe4 decreases the catalytic activity to about half. Several functional groups are tolerated in this catalytic reaction, including Br, OMe, Me, CF_3_, CN functionalities. Disubstituted alkynes are also converted (Scheme 17b), though the conversion rate is significantly slower than that of terminal alkynes and full conversion is not achieved. Likewise, alkyl-substituted alkynes are converted only moderately (60–80% after 24 h). In all cases, the semi-hydrogenation is highly selective towards the *Z*-isomer with *Z*/*E* ratios between 4 : 1 and >99 : 1.

**Scheme 18:**
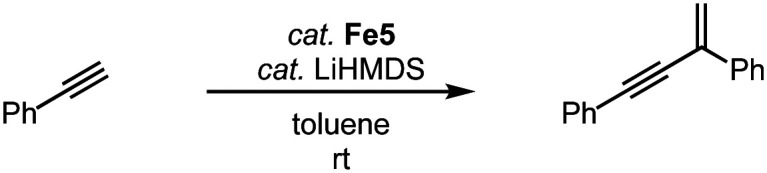
Geminal dimerization of phenylacetylene using iron piano-stool complex Fe5.

The two-legged piano-stool iron complex Fe5, developed by Song and coworkers, lacks the CO ligand when compared to complex Fe2. This complex is catalytically active in the geminal dimerization of phenylacetylene.^90^ Reacting phenylacetylene with 3 mol% catalyst and LiHMDS at room temperature affords the geminal enyne product selectively in 90% yield after 1 h and essentially quantitatively after 3 h (Scheme 18). Comparable yields are obtained with analogous iron complexes containing an imidazole-derived carbene ligand.

**Scheme 19:**
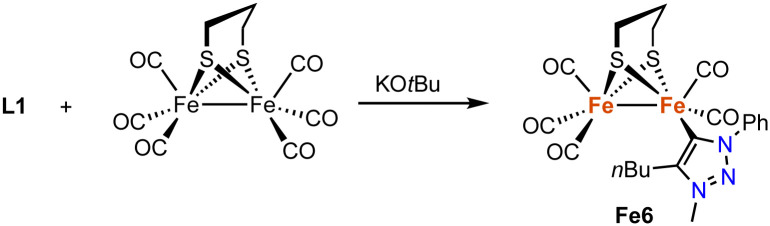
Synthesis of a diiron hydrogenase mimic featuring a monodentate trz ligand.

In addition, a range of non-piano stool trz iron complexes have been prepared and investigated for catalytic applications. For example, the diiron hydrogenase mimic Fe6 with a trz ligand is accessible by deprotonation of the triazolium salt L1 (R = Ph, R′ = *n*Bu, X = OTf) with KO*t*Bu in the presence of hexacarbonyldiiron propanedithiolate (Scheme 19).^91^ Complex Fe6 is the only trz complex with iron in the formal +1 oxidation state. It features a Fe–C_trz_ distance of 1.904(2) Å, comparable to other mono-NHC diiron complexes, yet significantly shorter than in piano-stool iron(ii) trz compounds. The complex is redox active with a quasi-reversible one-electron oxidation at +0.021 V and an irreversible two-electron reduction at −2.29 V (*vs.* Fc^+/0^). The complex is inactive in proton reduction independent of the amounts of acid present.

**Scheme 20:**
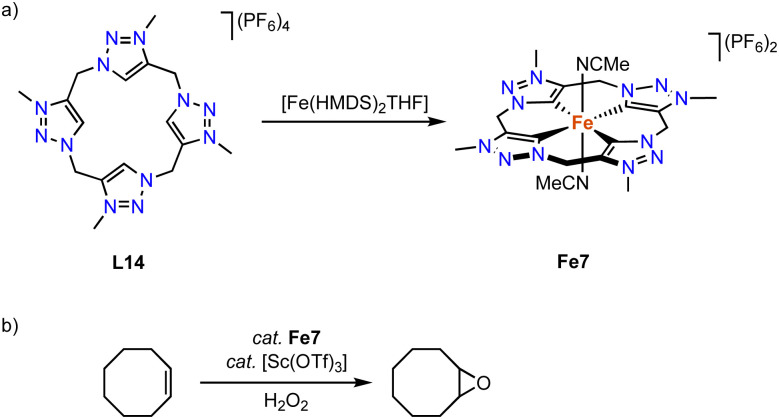
(a) Synthesis of iron complex Fe7 containing a macrocyclic tetrakis(trz) ligand; (b) epoxidation of cyclooctene catalyzed by Fe7.

Kühn and coworkers have synthesized an iron complex with a trz-based macrocyclic ligand.^92^ Treatment of ligand precursor L14 with two equivalents [Fe(HMDS)_2_(THF)] in MeCN at −40 °C, in which the HMDS ligands act as internal bases, yields complex Fe7 (Scheme 20) together with [Fe(MeCN)_6_](PF_6_)_2_, as the iron salt is used in excess. The iron center in this complex is distorted octahedral with an equatorial tetradentate macrocyclic ligand and two axially coordinating MeCN ligands. The confined ligand coordination results in Fe–C_trz_ bonds around 1.93(1) Å, which is significantly shorter than the bond lengths in mono and bidentate iron(ii) trz complexes with Fe–C_trz_ > 1.98 Å. The complex is electrochemically active and features a fully reversible one-electron oxidation at −0.34 V (*vs.* Fc^+/0^). The low potential of this putatively metal-centered process is rationalized by the strongly σ-donating properties of the four trz ligands. In comparison, the same oxidation process of the imidazole analogue is cathodically shifted by almost 0.5 V and occurs at +0.15 V (*vs.* Fc^+/0^).^93^ The facile oxidation of Fe7 promotes catalytic epoxidation reactions. At 0.5 mol% loading and in the presence of 10 mol% Sc(OTf)_3_ as additive and H_2_O_2_ as terminal oxidant, *cis*-cyclooctene is oxidized to cyclooctene oxide in 93% yield and with 97% selectivity. The TOF in the first 10 s of the reaction is very high, 41 000 h^−1^, and similar to benchmark catalysts.^94,95^

**Scheme 21:**
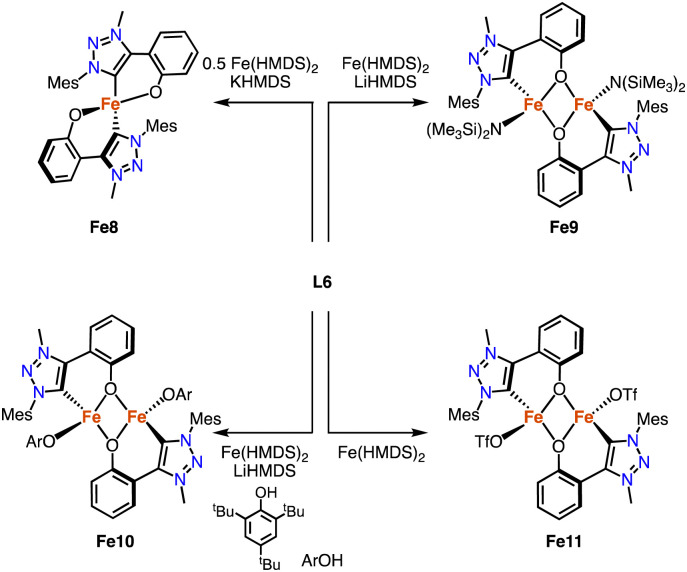
Synthesis of the homoleptic *C*,*O*-chelating iron trz complex Fe8, and the dimetallic complexes Fe9-Fe11.

Deprotonation of the phenol-substituted triazolium salt L6 (R = Mes) followed by addition of 0.5 equivalents [Fe(HMDS)_2_] yields the homoleptic iron complex Fe8 containing a *C*,*O*-bidentate chelating trz-phenoxy ligand (Scheme 21).^96^ The Fe–C_trz_ distance is 2.0406(12) Å, and the complex has a high-spin pentet electronic configuration in both the solid state and solution as determined by SQUID and Evans' method (SQUID = superconducting quantum interference device). Heteroleptic dimeric iron complexes were formed when using 1 rather than 0.5 equivalent [Fe(HMDS)_2_].^97^ The ancillary ligand varied by addition of either LiHMDS (Fe9) or ArOH (Fe10). In the absence of any additive, the OTf anion of ligand L6 is bound to form Fe11. The structures of Fe9 and Fe10 were confirmed by crystallographic analysis and show a Fe_2_O_2_ diamond core with bridging phenolates. The Fe–C_trz_ distances in these complexes are 2.129(4) and 2.0635(17) Å, respectively, featuring the longest Fe–C_trz_ bonds known to date.

**Scheme 22:**
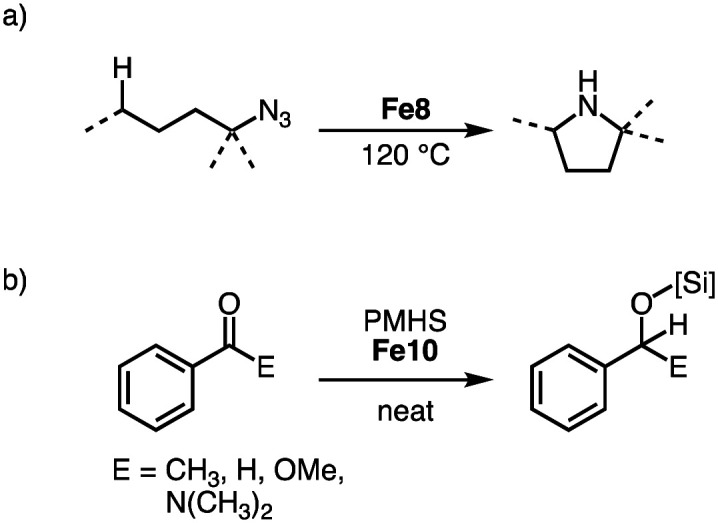
(a) Intramolecular C–H amination of tertiary alkylazides catalyzed by Fe8; (b) hydrosilylation of various carbonyl substrates with PMHS catalyzed by Fe9.

Reacting tertiary alkylazides in the presence of complex Fe8 at 120 °C results in catalytic C–H amination to afford 5-membered N-heterocycles (Scheme 22a). Complex Fe8 is the first iron catalyst for this transformation that does not require the addition of Boc_2_O to prevent product inhibition. This behavior is attributed to the steric congestion around the iron center imparted by the bulky trz ligand. In line with this model, the catalyst shows an unprecedented longevity with 7600 TONs, an order of magnitude higher than other catalyst that use organic azides for C–H amination. The beneficial role of the trz ligand is underpinned when comparing the performance of iron precursors [Fe(HMDS)_2_] and FeI_2_, which accomplish maximum TONs of only 180 and 370, respectively.^98–100^ A broad range of C–H bonds are aminated with Fe8, and the reaction tolerates aryl ether, bromide, and thiophene functional groups. Competition experiments indicate a high selectivity towards formation of 5-membered heterocycles, and kinetic studies imply a unique iron dimer resting state and rate-limiting N_2_ loss from the coordinated organoazide. Variation of the *N*-substituent in complex Fe8 indicate identical kinetics and confirm the half-order in iron, thus implying that the dimer resting state is more general for this class of catalysts.^101^

Both homoleptic Fe8 and bimetallic Fe9-Fe11 are active in the hydrosilylation of acetophenone.^97^ Using 10 ppm of Fe10 with PhSiH_3_ as silylating agent affords essentially quantitative yields at room temperature after 14 d, corresponding to 48 000 TON per iron center (Scheme 22b). The catalysts are also effective when using the less reactive silane polymethylhydrosiloxane (PMHS), a waste product from the silicon industry. Comparison of the different Fe complexes at 0.05 mol% loading at room temperature and under neat conditions reveals Fe9 as the fastest of the series with quantitative conversion within 5 h. Complexes Fe8 and Fe10 require 9 h for similar conversions. Reducing the loading of Fe9 to 10 ppm is possible and yields 36 000 TON per iron center, albeit only after 14 d. This catalyst performance is unprecedented for base metal catalyzed hydrosilylation with PMHS. Also, aldehydes and more challenging substrates like esters and amides are hydrosilylated with Fe10, however elevated temperatures and higher catalyst loadings are required for reaching useful conversions.

**Scheme 23:**
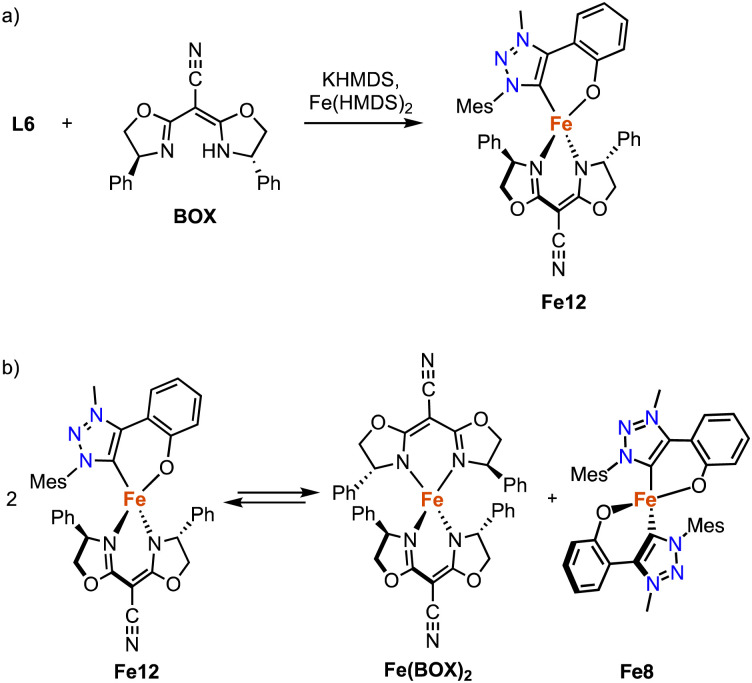
(a) Synthesis of the heteroleptic *C*,*O*-chelating iron trz complex Fe12; (b) ligand redistribution equilibrium observed with Fe12, demonstrating the lability of the *C*,*O*-bidentate ligand at an Fe(ii) center.

The strategy for the synthesis of monocarbene complexes with L6 has been expanded to bidentate rather than monodentate ancillary ligands. Using a chiral bisoxazoline (BOX) ligand together with L6 and Fe(HMDS)_2_ affords the heteroleptic monomeric complex Fe12 (Scheme 23a).^102^ Stability studies indicate a ligand redistribution equilibrium between Fe12 and the corresponding homoleptic complexes Fe8 and Fe(BOX)_2_ (Scheme 23b). This behavior demonstrates the lability of the Fe–C_trz_ bond even within a strongly chelating framework. Using Fe12 for asymmetric intramolecular C–H amination yields only a racemic mixture, which is commensurate with the formation of the achiral Fe8 as catalytically most competent species in the equilibrium, while Fe(BOX)_2_ is catalytically silent.

**Scheme 24:**
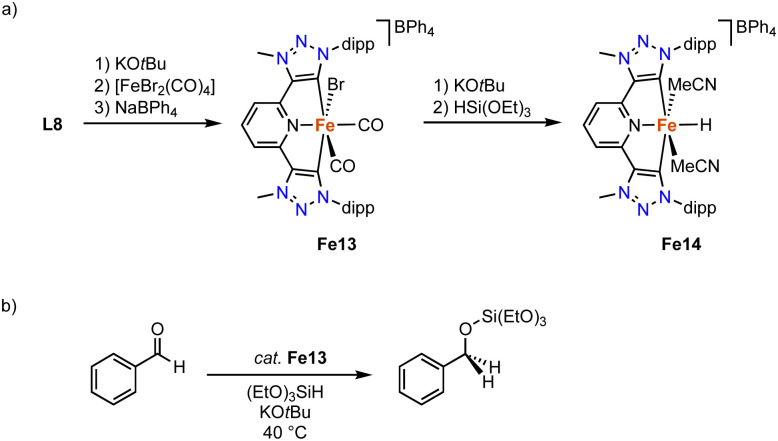
(a) Synthesis of the pincer trz iron complex Fe13 and the putative hydride Fe14; (b) catalytic hydrosilylation of benzaldehyde with Fe13.

Complex Fe13 containing pincer-type trz ligand is accessible by deprotonation of ditriazolium salt L8 (R = dipp, X = BF_4_)^103^ with KO*t*Bu to generated the carbene *in situ*, and subsequent coordination to [FeBr_2_(CO)_4_] (Scheme 24a).^104^ The resulting complex is unstable, yet addition of NaBPh_4_ affords the stable diamagnetic pincer complex Fe13. The Fe–C_trz_ bond lengths in the octahedral complex are 1.951(3) Å. The two distinct *cis*-coordinated CO ligands produce symmetric and asymmetric vibration bands at 2040 and 1985 cm^−1^, respectively. Heating complex Fe13 to 100 °C induces decoordination of both CO ligands to presumably yield a four coordinate iron complex. Complex Fe13 is catalytically activity in the hydrosilylation of benzaldehyde with (EtO)_3_SiH at 40 °C. At 5 mol% of catalyst loading and in the presence of 12.5 mol% KO*t*Bu, quantitative yields are obtained within 24 h, while only 40% are achieved in the absence of base (Scheme 24b). Stoichiometric experiments indicate that addition of (EtO)_3_SiH and KO*t*Bu to Fe13 yield an unstable iron hydride complex Fe14 according to IR- and Mössbauer spectroscopies (Scheme 24a). DFT calculations suggest a synergy between the base and the silane to facilitate bromide abstraction and hydride coordination.

**Scheme 25:**
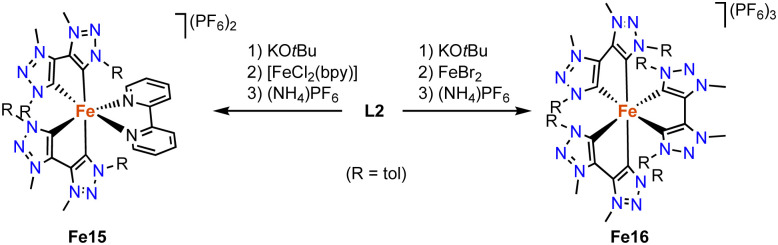
Synthesis of the heteroleptic iron(ii) complex Fe15 and the homoleptic octahedral iron complex Fe16 with all six coordination sites occupied by trz ligands.

Wärnmark and Sundström discovered in 2015 that replacing the 2,2′-bipyridine (bpy) ligands on iron by bis(trz) ligands improves the photophysical properties of the complex. The heteroleptic octahedral iron complex Fe15 containing two bidentate trz ligands and one bpy ligand is formed *via* deprotonation of the ditriazolium salt L2 (R = tol, X = Br) with KO*t*Bu, addition of [FeCl_2_(bpy)], and counterion exchange with (NH_4_)PF_6_ (Scheme 25).^105^ Structural characterization of the analogue with bromide counterions reveals the expected octahedral geometry with four trz donor sites, and Fe–C_trz_ bond lengths in the 1.96–2.02 Å range, similar to other Fe^II^ trz complexes. Complex Fe16, *viz* the homoleptic version of complex Fe15 and comprised exclusively of trz donors around the iron center, is synthesized by double deprotonation of 3 equivalents of ligand precursor L2 (R = tol, X = Br) and metalation with FeBr_2_, followed by counterion exchange (Scheme 25).^106^ The resulting complex features a trivalent iron center, presumably induced by the strong donor properties of the six trz ligands around the iron center. The Fe–C_trz_ bond lengths are slightly shorter, between 1.95 and 1.98 Å. Several spectroscopic and computational techniques indicate the complex to feature a low-spin Fe^III^ center. The complexes undergo a reversible electron transfer at −0.35 V (Fe15) and −0.58 V (Fe16; *vs.* Fc^+/0^), which according to EPR and UV-Vis spectroscopies is due to a Fe^III^/Fe^II^ redox couple. The anodic shift for Fe16 illustrates the strong electron donor properties of the trz ligands. Complex Fe15 features a triplet metal-to-ligand charge transfer, ^3^MLCT, with a remarkably long lifetime of 13 ps, the longest photoexcited life-time for iron at that time.^107^ Complex Fe16 reveals altered photophysical properties and is the first iron complex that displays photoluminescence upon photoexcitation, with quantum yields of 3 × 10^−4^ in MeCN at room temperature and 8 × 10^−4^ at 100 K. The emission originates from a rare^108,109^ doublet excited ligand-to-metal charge transfer (^2^LMCT), with a lifetime of 107 ps at room temperature and 430 ps at 100 K. This is one of the longest lifetimes in iron-based photoluminescence.^110^

**Scheme 26:**
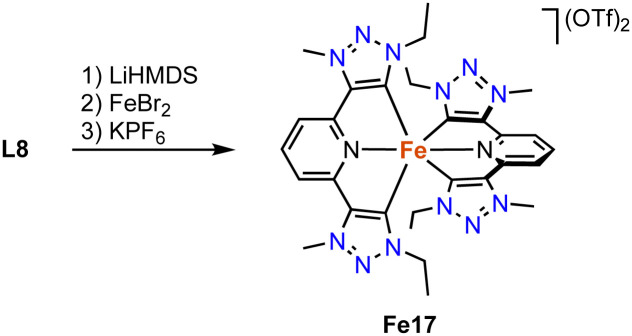
Synthesis of the homoleptic bis(trz-pincer) iron complex Fe17.

An alternative trz ligand design for photophysical properties is based on a *C*,*N*,*C*-pincer type trz-pyr-trz ligand motif. The homoleptic iron complex Fe17 is synthesized by standard procedures, *i.e.* deprotonation of the ditriazolium salt L8 (R = Et, X = OTf) with a strong base (LiHMDS) and subsequent FeBr_2_ coordination and counterion exchange (Scheme 26).^111^ The transient absorption (TA) spectrum, measured by ultrafast pump–probe spectroscopy, reveals two negative bands at around 600 and 420 nm, in line with the measured UV-Vis absorption spectrum. In addition, the complex features two excited state absorptions around 455 nm and in the red spectral region (>300 nm). Time-dependent measurements show a fast ground state bleaching within 50 ps. These measurements combined with decay-associated spectra indicate that the decay of the excited state occurs through three distinct intermediates. Initially, the singlet excited metal-to-ligand charge transfer (^1^MLCT) state undergoes an ultrafast intersystem crossing to a vibrationally hot triplet excited ^3^MLCT state, most of which decays to the triplet ^3^MC state within 0.1 ps. A minor part undergoes a vibrational redistribution within 0.1 ps to obtain a relaxed triplet excited ^3^MLCT state, which also decays to the triplet excited ^3^MC state, but much slower in 8.7 ps. Lastly, the triplet excited MC state decays to the original ground state iron complex.

### Cobalt

**Scheme 27:**
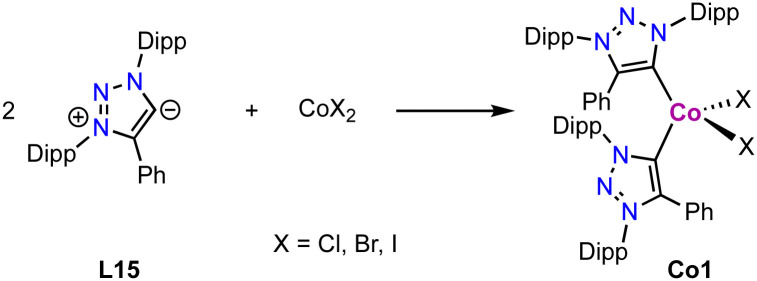
Synthesis of tetrahedral bis trz cobalt complexes Co1 with different halides.

Cobalt(ii) trz complexes are accessible by methods similar to those of iron. Thus, metalation of the free carbene L15 derived from its triazolium salt with 0.5 equivalent CoX_2_ (X = Cl, Br or I) yields the corresponding tetrahedral bis(trz) cobalt complex Co1 (Scheme 27).^112^ These complexes display a distorted tetrahedral geometry with an angular distortion value τ_4_ of 0.89, irrespective of the coordinated halide. The magnetic properties of the complexes, determined by SQUID measurements, show some dependence on the halide: while the chloride complex displays a constant *χ*_M_*T* value above 25 K, the *χ*_M_*T* values of the bromide and iodide analogues are temperature-dependent. At room temperature, the *χ*_M_*T* values of the complexes (2.37, 2.63 and 2.79 cm^3^ mol^−1^ K for X = Cl, Br and I, respectively) are higher than the spin-only value for Co(ii) in the quartet spin state, indicating contributions of higher laying spin states towards the ground state. This model is supported by the increased contribution from Cl to Br and I, as the d-orbital splitting decreases in this sequence. The same trend is also observed in the UV-Vis absorption spectra of these complexes.

**Scheme 28:**
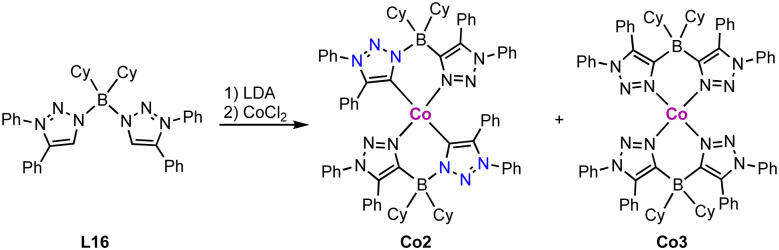
Synthesis of a borate based bis(trz) cobalt complex Co2 and its C–N isomerized product Co3 containing only N-bound triazole ligands.

Homoleptic square planar Co(ii) complexes bearing two borate-linked bis(trz) ligands are accessible from the corresponding bis(triazolium)borate L16. Deprotonation and metalation with CoCl_2_ affords an inseparable mixture of cobalt complexes Co2 and Co3 (Scheme 28).^113^ Complex Co2 displays a C/N isomerization between the cobalt and the boron for two trz fragments due to the weak B–N bond in the ligand, resulting in *N*- rather than *C*-coordination of the triazole-based ligand. In complex Co3, all trz fragments have undergone this isomerization, and the complex is a coordination compound without a carbene ligand. Reaction of ligand precursor L16 with LiTMP (TMP = 2,2,6,6-tetramethylpiperidyl) induces faster isomerization and indicates a low stability of the ligand. A similar shift of the N–CH_3_ substituent to the carbenic position of free triazolylidenes has been demonstrated earlier by Bertrand and coworkers.^19^

**Scheme 29:**
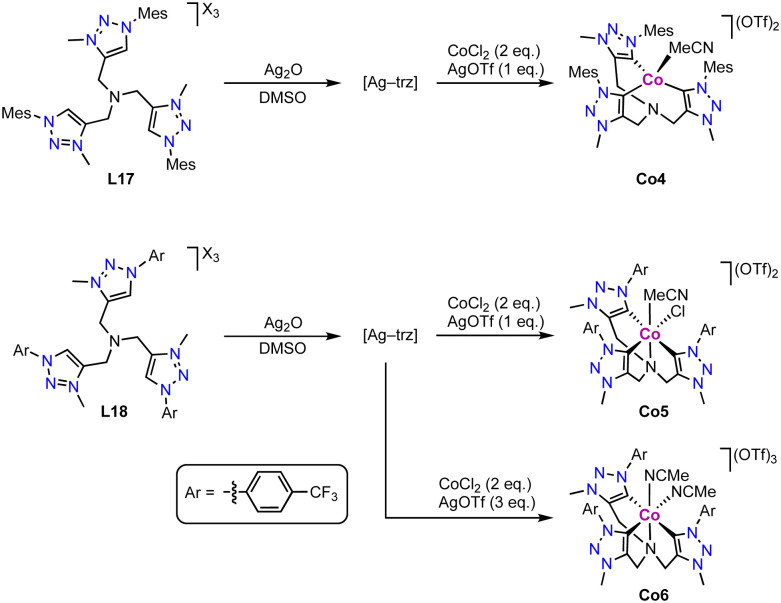
Synthesis *via* transmetalation of the tris(trz) Co(ii) complex Co4 and Co5, and analogous Co(iii) complex Co6.

The tripodal tris(trz) cobalt(ii) complex Co4 is prepared *via* transmetalation from the corresponding silver complex, which in turn is obtained upon reaction of the tris(triazolium) salt L17 with Ag_2_O (Scheme 29).^114^ Interestingly, the bridging nitrogen remains uncoordinated upon complexation and the ligand adopts a tridentate bonding mode. The cobalt coordination sphere is completed by an external MeCN ligand. The three coordinated trz units feature an average Co–C_trz_ bond length of 2.016(4) Å. Remarkably, modification of the trz wingtip group from Mes to *p*-CF_3_-phenyl as in ligand L18 leads to different product formation. Under identical reaction conditions, the octahedral cobalt(iii) complex Co5 is obtained, in which the tripodal ligand binds in a tetradentate coordination mode, including the three trz and the bridging nitrogen. The oxidation from Co^II^ to Co^III^ is entailed by AgOTf. The different coordination modes of ligand L17 in complexes Co4 and Co5 are presumably a consequence of the larger steric demand of the Mes wingtip group compared to the *p*-CF_3_-phenyl group. The Co–C_trz_ bond lengths for the mutually *trans* positioned trz units are 1.98(1) Å in Co5, and much shorter (1.871(8) Å) for the trz *trans* to chloride. In the presence of excess AgOTf, the tris-cationic complex Co6 with two coordinated MeCN ligands is formed.

**Scheme 30:**
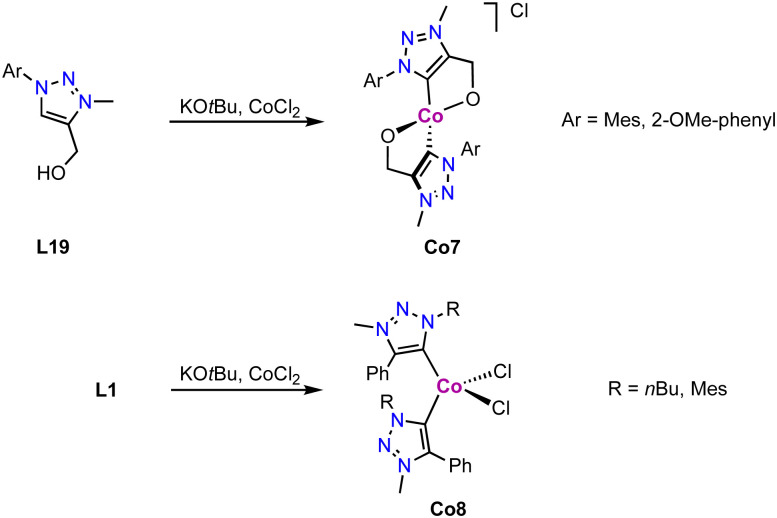
Synthesis of cobalt trz complexex Co7 comprised of a *C*,*O*-chelating trz ligand and Co8 with monodentate trz ligands.

Tetrahedral cobalt(iii) complexes Co7 containing *C*,*O*-bidentate chelating trz ligands are obtained upon deprotonation of the alkoxy-functionalized triazolium salt L19 and subsequent metalation with CoCl_2_ (Scheme 30).^115^ The complexes have a magnetic moment of 3.1 μ_B_, indicative of an intermediate spin (*S* = 1) Co^III^ center, suggesting air-mediated one electron oxidation during coordination. Performing the same reaction with monodentate ligand precursor L1 (R = *n*Bu, Mes; R′ = Ph; X = OTf) affords complex Co8 akin to Co1 (Scheme 30). The magnetic moment, measured by Evans' method, *μ*_eff_ = 3.8 μ_B_ indicates an intermediate spin (*S* = 3/2) as expected for a Co^II^ center. The oxidation of the metal center in Co7 but not in Co8 is presumably a consequence of the strong alkoxide and trz donor set in the former complex, and ensuing easy oxidation of the cobalt center. Interestingly, the measured magnetic moment of Co8 is equal to the spin only value for a quartet spin state, indicating no contribution of higher laying spin states towards the ground state. This is in agreement with the magnetic properties of the structurally related cobalt complexes Co1.^112^

**Scheme 31:**
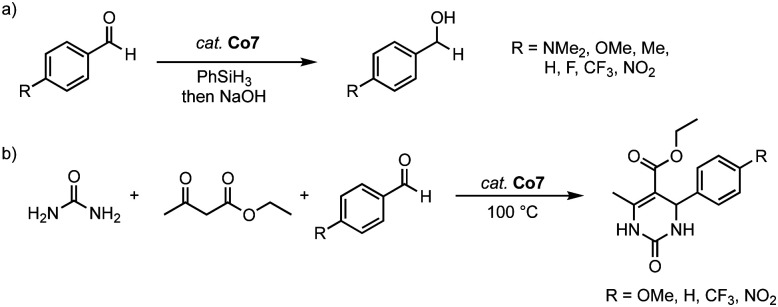
(a) Catalytic hydrosilylation of substituted benzaldehydes using cobalt complexes Co7. (b) Three-component Biginelli reaction catalyzed by the same cobalt complex Co7.

The homoleptic cobalt(iii) complex Co7 is catalytically active in both the hydrosilylation of carbonyl groups and dihydropyrimidinone synthesis. Thus, 2 mol% of the complex catalyzes the hydrosilylation of 4-methylbenzaldehyde in up to 96% yield (R = 2-OMe-phenyl) after 4 h at 40 °C. In contrast, the monodentate cobalt(ii) complexes Co8 afford the hydrosilylation product only in 33% yield under identical conditions, irrespective of the wingtip group (Scheme 31a). This large discrepancy is attributed to the different oxidation states of the catalysts and the potential of the *C*,*O*-ligand to act as a non-innocent ligand to facilitate Si–H bond activation. Complex Co7 displays an excellent functional group tolerance in hydrosilylation catalysis, with the exception of the nitro group, which deactivates the catalyst. Kinetic analyses demonstrate an inverse Hammett correlation, suggesting the build-up of a positive charge during the turnover-limiting step. Ketones are not hydrosilylated with complex Co7, thus limiting the scope of the catalysis, yet introducing some selectivity of the process. At maximum performance, the cobalt catalyst reaches TOF = 180 h^−1^ and TON = 400, considerably lower values than related trz complexes based on iron (see above) or nickel (see below). Noteworthy, complex Co7 is also catalytically active in the Biginelli reaction for the synthesis of dihydropyrimidinone (Scheme 31b). With benzaldehyde as the model substrate, complex Co7 shows high activity and gives the arylated dihydropyrimidinone in 99% yield after only 1 h at 100 °C, with a TOF_50_ = 200 h^−1^. Methoxy and CF_3_-groups are tolerated by the catalyst and give essentially quantitative yields after 90 and 15 min, respectively. As observed with the hydrosilylation, the nitro group is inhibiting catalytic turnover, resulting in only poor 11% yield.

### Nickel

**Scheme 32:**
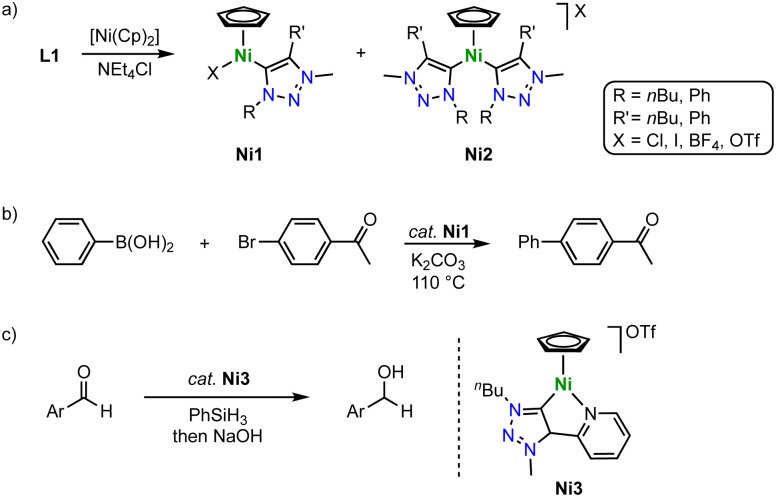
(a) Synthesis of trz nickel Cp complexes Ni1 and Ni2*via* direct C–H bond activation; (b) Suzuki–Miyaura cross-coupling catalyzed by complexes Ni1; (c) hydrosilylation of aldehydes catalyzed by complex Ni3, prepared similarly to Ni1.

Half-sandwich nickel(ii) Cp complexes containing a trz ligand are formed in the absence of a base, thus contrasting complexation routes with Mn, Fe, Co and other 3d metals. According to a method developed for imidazolium salt nickelation, exposure of the triazolium salt L1 with various substituents and counterions to [Ni(Cp)_2_] induces triazolium C–H activation and affords complexes Ni1 with a variety of trz wingtip groups (Scheme 32a).^116^ The reaction typically affords a mixture of mono- and dicarbene Ni(Cp) complexes Ni1 and Ni2, with ratios depending on the temperature, the anion, and the reaction time. Kinetic analysis of the metalation reaction indicates that the mono(trz) complex Ni1 is a precursor to the bis(trz) complex Ni2, which implies a carbene transfer between nickel centers. The crystal structure revealed similar Ni–C_trz_ bond lengths for all nickel complexes around 1.88(1) and 1.893(1) Å. The mono(trz) nickel complexes Ni1 are highly active catalysts for Suzuki–Miyaura cross-coupling of boronic acids and aryl halides with initial TOF up to 250 h^−1^ (Scheme 32b). However, time-conversion profiles demonstrate high activity only for the first few minutes. After a short period, catalyst deactivation becomes significant, which is attributed to an insufficient stability of the Ni–C_trz_ bond towards boronic acids. Therefore, full conversion is only achieved upon repetitive addition of Ni1 and boronic acid substrate.

These complexes are also excellent catalysts for the hydrosilylation of aldehydes with PhSiH_3_.^117^ Especially the introduction of a chelating pyridyl group on the trz ligand (Ni3) raises catalytic activity and provides TOFs up to 23 000 h^−1^ (Scheme 32c). Complete hydrosilylation of aldehydes is accomplished at low 0.05 mol% catalyst loading, and maximum TONs around 6000 are accessible. This performance exceeds most of the iron-based trz hydrosilylation catalysts except for Fe10 and outperforms the cobalt trz catalysts by two orders of magnitude.

**Scheme 33:**
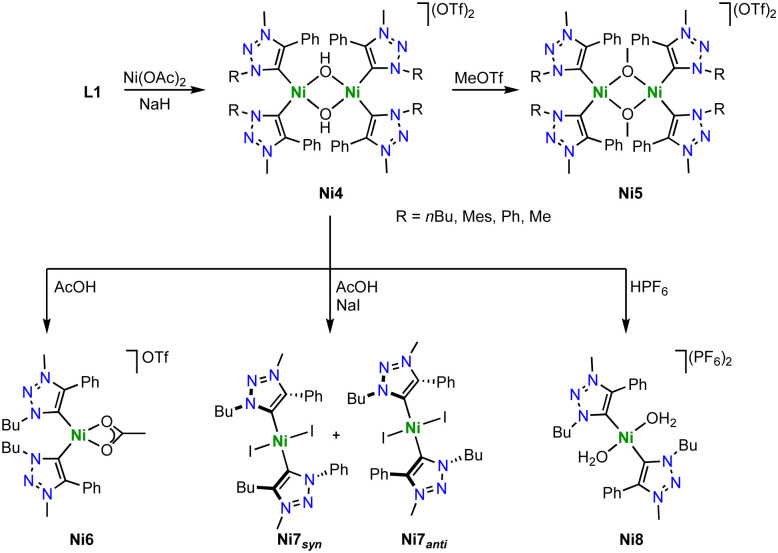
Synthesis of the hydroxide-bridged dimetallic nickel complex Ni4 with two monodentate trz ligands and the reactivity of this complex towards MeOTf and strong acids to form bimetallic Ni5 as well as monometallic complexes Ni6–8.

With Ni(OAc)_2_ as metal precursor and in presence of NaH as a base, the triazolium salts L1 with various substituents reacts to form hydroxide-bridged dimetallic nickel complexes Ni4 bearing monodendate trz ligands (Scheme 33).^118^ In the solid state, the nickel center in Ni4 is square planar and the Ni–C_trz_ distances are in the 1.875(2)–1.929(2) Å range. Contrary to its imidazolium counterpart, Ni4 does not exchange the bridging hydroxide ligand with alkoxides upon exposure to MeOH or EtOH. This reactivity difference suggests a decreased nucleophilicity of the bridging hydroxide ligand due to the stronger σ-donor properties of the trz compared to imidazolylidene ligands.^30^ Addition of MeOTf to Ni4 methylates the bridging hydroxide ligands and yields the methoxide-bridged dinickel complexes Ni5 (Scheme 33). The dimetallic complexes are robust towards weak acids (p*K*_a_ > 6), though stronger acids (p*K*_a_ < 5) induce cleavage of the dimer. For example, reaction of Ni4 (R = *n*Bu) with AcOH affords the monometallic complex Ni6 with a bidentate coordinated acetate ligand. Performing the same reaction in the presence of NaI yields the *trans* bis(trz) complex Ni7 as a 3 : 1 mixture of *syn*/*anti*-isomers, that is, the same wingtip groups on the same or opposite side of the nickel square plane. These reaction patterns indicate that the most basic site in the dimetallic complex Ni4 is the bridging hydroxide and not the trz ligand. Only when exposed to strong acids such as HPF_6_, the aqua complex Ni8 is observed together with free ligand precursor L1, suggesting acid–base reactivity now also involving the trz ligand. Of note for further application, complex Ni4 does not show any reactivity towards a variety of bases, *e.g.* NaOMe, Na_2_CO_3_ or Et_3_N, demonstrating that neither the nickel center nor the bridging hydroxy group display pronounced acidic character.

**Scheme 34:**
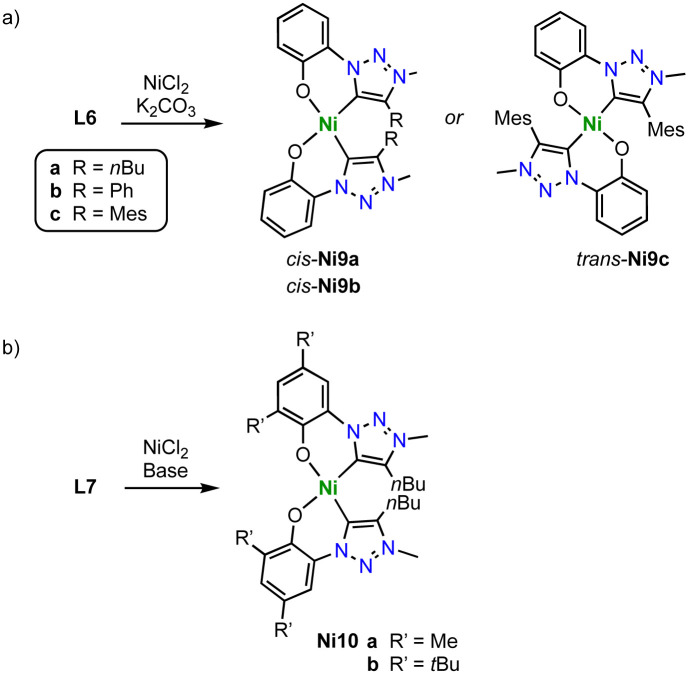
Synthesis of homoleptic *C*,*O*-chelating trz nickel complexes Ni9 and Ni10.

Monometallic *C*,*O*-chelating trz nickel complexes are obtained upon introduction of a phenol wingtip group to the triazolium scaffold as in ligand precursor L6 (Scheme 34a).^119^ Exposure of L6 (R = *n*Bu, Ph, Mes) to NiCl_2_ in the presence of K_2_CO_3_ yields the homoleptic nickel complexes [Ni(O^trz)_2_] Ni9a-c. With less bulky butyl and phenyl substituents on the trz heterocycle, the electronically favored *cis*-isomer, *cis*-Ni9a-b is formed, though steric repulsion of the substituents distorts the square-planar geometry markedly (*τ*_4_ ≥ 0.15). Bulkier mesityl substituent cannot be accommodated anymore in a *cis*-arrangement and instead, *trans*-Ni9c is isolated. In the *trans*-configuration, the square-planar geometry of the nickel complex is significantly less distorted (*τ*_4_ ≤ 0.02). Complexes Ni9a-c are active in electrocatalytic reduction reactions. For example, complexes *cis*-Ni9a-b catalyze proton reduction to form H_2_ in the presence of AcOH as proton source with *k*_obs_ = 440 and 300 s^−1^, respectively. The *trans*-complex Ni9c is more than an order of magnitude less active (*k*_obs_ = 10 s^−1^). In addition, the complexes electrocatalytical reduce CO_2_ to formate at −1.8 V (*vs.* normal hydrogen electrode, NHE). Addition of MeOH or H_2_O enhances the catalytic current considerably, in agreement with beneficial effects of a proton source. Analogous to proton reduction catalysis, *cis*-N9a is most active with *k*_obs_ = 280 s^−1^, while complex *trans*-Ni9c shows again only negligible activity, *k*_obs_ = 10 s^−1^. Analysis of the products reveals a unique selective towards formate, with no detectable amounts of CO as the typical product from CO_2_ reduction with 3d transition metal catalysts. faradaic efficiencies up to 68% are accessible after 8 h, with only traces of H_2_ from proton reduction pathways. Further optimization of the catalyst by increasing the ligand donor properties is accomplished by introducing methyl or *tert*-butyl groups in *ortho*- and *para*-position of the phenolate unit of ligand L7 (R = Me, *t*Bu; Scheme 34b). The corresponding nickel(ii) complexes Ni10, show an enhanced catalytic performance. Especially the *t*Bu substituents lead to higher rates *k*_obs_ = 370 s^−1^, and improved faradaic efficiency of 83%. This complex is not only the best performing catalyst of the trz series, but also outperforms by far analogues with imidazolylidene instead of trz ligand sites. Mechanistically, the necessity of the *cis*-arrangement of ligands and the need for an electron-rich nickel center suggest that the two oxygen donors in *cis*-Ni9a-b or its reduced form act as a proton sponge. Such proton chelation pre-organizes the proton for oxidative ligation to form the nickel hydride species that is required for CO_2_ hydrogenation to formate, rather than CO_2_ reduction to CO.

**Scheme 35:**
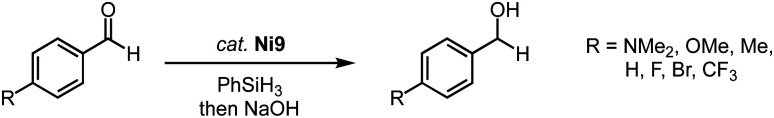
Hydrosilylation of *para*-substituted benzaldehydes catalyzed by complex Ni9.

Complexes Ni9a–c are also efficient catalysts for the hydrosilylation of substituted benzaldehydes (Scheme 35).^120^ A variety of functional groups are tolerated including halides, ethers, and amines, though again the nitro group is not compatible. Complex *cis*-N9a as the best hydrosilylation catalyst of this series reaches at 0.02 mol% loading and 60 °C reaction temperature TOF_max_ = 27 000 h^−1^ and at even lower catalyst loading (0.01 mol%) up to 7400 turnovers.

**Scheme 36:**
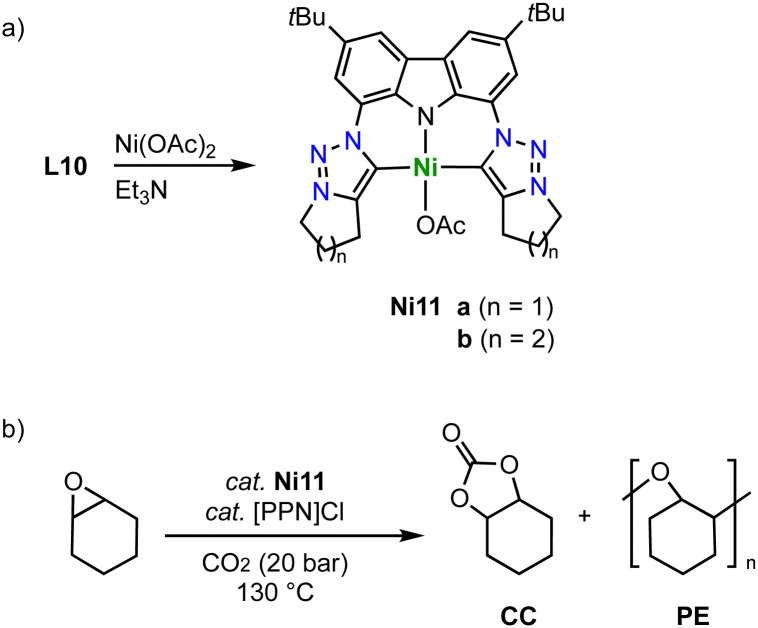
(a) Synthesis of a square planar nickel complex Ni11 with a trz pincer ligand; (b) catalytic coupling of CO_2_ and epoxides to carbonates catalyzed by Ni11.

Tridentate trz ligation to nickel is achieved when using pincer-type ligands. For example, metalation of the ditriazolium salts L10 (*n* = 1–2) with [Ni(OAc)_2_] in the presence of Et_3_N affords complexes Ni11 (Scheme 36a).^121^ The Ni–C_trz_ bond lengths in these complexes are 1.94(1) Å and thus longer compared to monodentate complexes. The complexes catalyze the cycloaddition of CO_2_ and cyclohexene oxide to form cyclic carbonates (Scheme 36b). Using 0.1 mol% catalyst and 0.1 mol% bis(triphenylphosphine)iminium chloride, [[PPN]Cl], as co-catalyst at 130 °C and 20 bar CO_2_, full conversion and exquisite selectivity is accomplished with complex Ni11a (*n* = 1). Complex Ni11b (*n* = 2) shows similar activity, though the selectivity is compromised and 7% polyether (PE) side product are formed. Absence of the co-catalyst or decreasing the CO_2_ pressure to 2 bar decreases the yield significantly and furthermore increases PE formation.

**Scheme 37:**
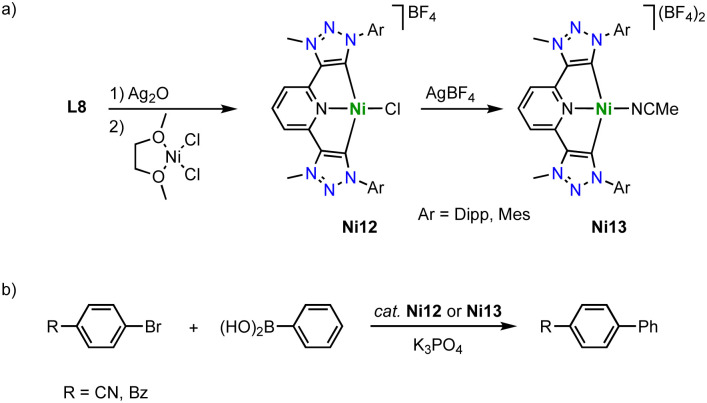
(a) Synthesis of square planar nickel pincer trz complexes Ni12 and Ni13; (b) Suzuki–Miyaura coupling with aryl bromides catalyzed by the nickel trz complexes Ni12 and Ni13.

Similar pincer-type trz nickel complexes are accessible by transmetalation. Reaction of the ditriazolium pincer ligand precursor L8 (R = Dipp, Mes; X = BF_4_) with Ag_2_O and subsequently with [NiCl_2_(dme)] yields the air-stable monocationic nickel complex Ni12 (dme = 1,2-dimethoxyethane; Scheme 37).^122,123^ Despite the formally cationic nickel center, the metal-bound chloride is readily abstracted upon reaction with AgBF_4_ to yield the dicationic nickel solvento complex Ni13, which is however unstable under aerobic conditions. The Ni–C_trz_ bond lengths in Ni12 are 1.905(2) and 1.909(2) Å and thus shorter than in the carbazole pincer complex Ni11. The complex is redox active and features an irreversible oxidation at +0.85 V (*vs.* Fc^+/0^) for R = Dipp and and at +0.70 V for R = Mes, and a second irreversible oxidation around +1.3 V. The complexes show moderate activity in Suzuki–Miyaura cross-coupling catalysis, reaching 74% yield after 8 h at 5 mol% loading of Ni12 (R = Dipp; Scheme 37b). The solvento complex Ni13 is similarly active, while the analogue containing imidazolylidene instead of trz donor sites shows better performance.

### Copper

**Scheme 38:**
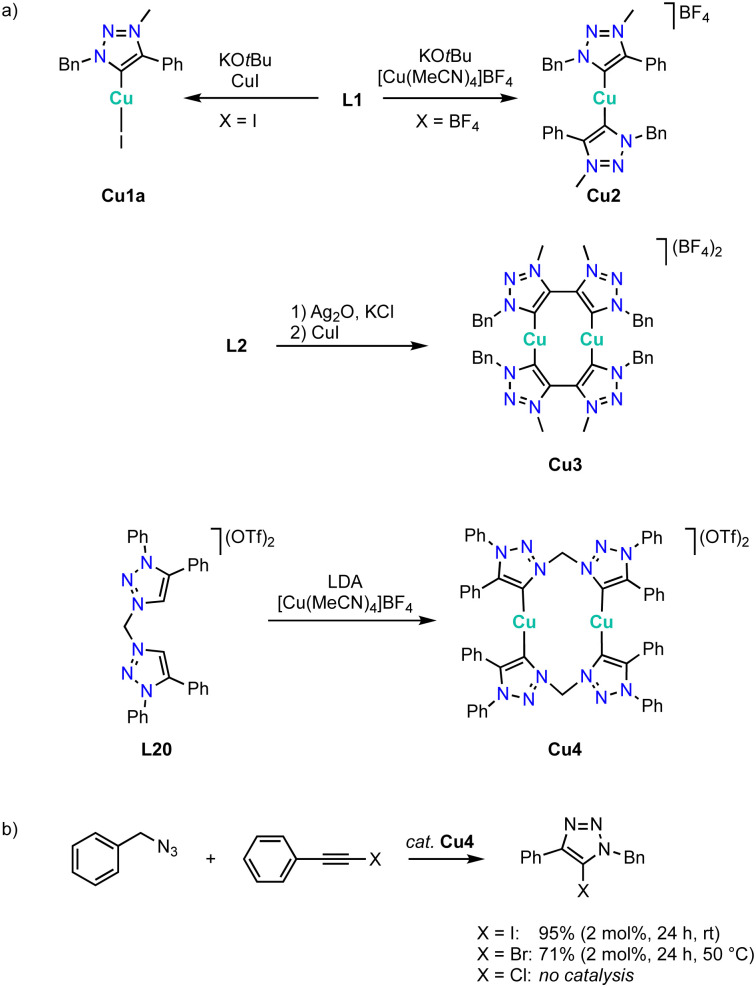
(a) Synthesis of copper(i) complexes Cu1 and Cu2 with monodentate trz ligands, and bimetallic complexes Cu3 and Cu4 from bis(trz) ligands; (b) halo-click reaction catalyzed by copper trz complexes Cu1a and Cu4.

Copper complexation with trz ligands follows the trend observed with Arduengo-type NHCs^124–127^ and yields mono(trz) complexes Cu1a in the presence of halides such as ligand precursor L1 (R = Bn, R′ =Ph) with an iodide counterion, and cationic bis(trz) complexes Cu2 in the presence of non-coordinating counterions (Scheme 38a).^128–131^ Bimetallic copper complexes are obtained from bidentate trz ligand precursor L2 (R = Bn, X = BF_4_), as the bridge between the two trz prevents a *trans* disposition of the carbenes. While a transmetalation approach using Ag_2_O and CuI provides access to complex Cu3, ligand precursor L20 requires a deprotonation by LDA and metalation using [Cu(MeCN)_4_]BF_4_ to yield the corresponding copper complex Cu4 cleanly. The trz copper complexes catalyze the halo-click reaction between iodoethynyl benzene and benzyl azide (Scheme 38b). The cationic complex Cu4 is more active than the neutral mono(trz) analogue Cu1a and reaches 92% yield at 5 mol% catalyst loading within 24 h at room temperature. Bromide substrates such as bromoethynyl benzene require slightly elevated temperatures. At 50 °C, bimetallic Cu4 is more active than the monometallic analogues and reaches 71% conversion after 24 h with 2 mol% copper loading. In contrast, chloroethynyl benzene is not converted by these complexes as the yields are identical to those from blank reactions.

**Scheme 39:**
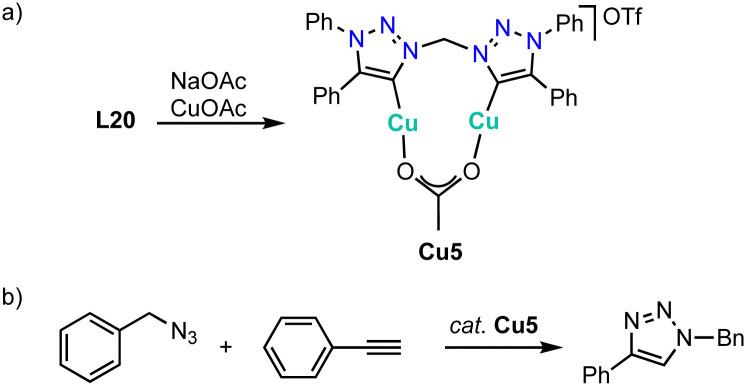
(a) Synthesis of the bimetallic copper complex Cu5 with bridging bis(trz) and OAc^−^ ligands; (b) click reaction between phenyl acetylene and benzyl azide catalyzed by complex Cu5.

Metalation of ligand precursor L20 with CuOAc in the presence of NaOAc affords the monocationic Cu_2_ complex Cu5 in which the anionic acetate ligand bridges both copper centers (Scheme 39a).^132^ This complex catalyzes the classic CuAAC click reaction between phenyl acetylene and benzyl azide (Scheme 39b). Kinetic studies show a first order dependance on the azide substrate and a broken order in catalyst (0.87), which has been attributed to a reversible dimerization of the bimetallic copper complex in the resting state. A substrate scope indicates catalyst instability in the presence of protic or bulky substrates.

**Scheme 40:**
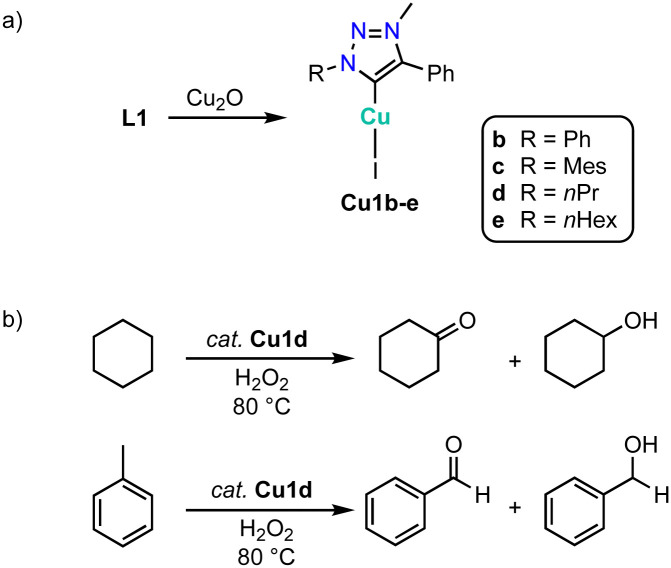
(a) Synthesis of copper trz complexes Cu1b–e*via* an internal base approach; (b) cyclohexane and toluene oxidation catalyzed by complex Cu1d.

An alternative method to generate complexes Cu1 from triazolium salts L1 involves the use of Cu_2_O instead of an exogenous base (Scheme 40a).^133^ Complexes Cu1b-e are moisture sensitive in solution and gradually decompose to L1 within 24 h. The complexes are moderately activity in the oxidation of cyclohexane with H_2_O_2_ at 80 °C (Scheme 40b). For example, complex Cu1d reaches at 1 mol% loading up to 39% conversion to afford a 1 : 2 mixture of cyclohexanone and cyclohexanol. Oxidation of *n*-octane proceeds with a lower 22% conversion, though the selectivity changes to 80% octanones. Similarly, toluene oxidation is less prolific with 11% conversion, yet the product mixture is composed predominantly of benzaldehyde and only minor quantities of benzyl alcohol. In agreement with these latter selectivities, complex Cu1d shows good catalytic activity in alcohol oxidation of benzylic alcohols (60–90% conversion), while aliphatic alcohols are oxidized less efficiently (30–70% conversion).

**Scheme 41:**
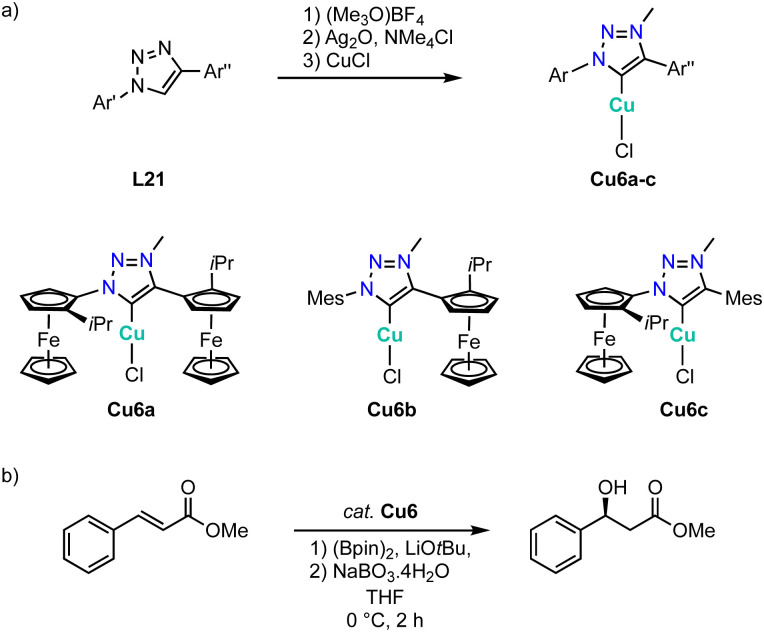
(a) Synthesis of planar-chiral copper trz complexes Cu6a–c with planar chiral ferrocene wingtip groups; (b) asymmetric borylation of methyl cinnamate catalyzed by copper trz complexes Cu6a–c.

While essentially all trz ligand precursors are achiral, Haraguchi and Fukuzawa have developed routes to the first chiral trz complexes with 3d transition metals.^134^ Methylation of triazoles L21 containing one or two planar-chiral ferrocenyl wingtip groups by (Me_3_O)BF_4_ followed by metalation using Ag_2_O and subsequent transmetalation with CuCl affords the chiral copper complexes Cu6a–c (Scheme 41a). Single crystal structure analysis of complexes Cu6b and Cu6c shows the expected linear coordination geometry. All three complexes are active in the asymmetric borylation of cinnamic esters (Scheme 41b). While yields are moderate (45–52%), the asymmetric induction is interesting. Thus, complexes Cu6b and Cu6c containing only one chiral substituent produce the β-hydroxyester in 52% ee, while complex Cu6a with two chiral substituents induces a higher 60% ee. Of note, this is the first catalytic process with chiral trz 3d complexes that shows asymmetric induction.

**Scheme 42:**
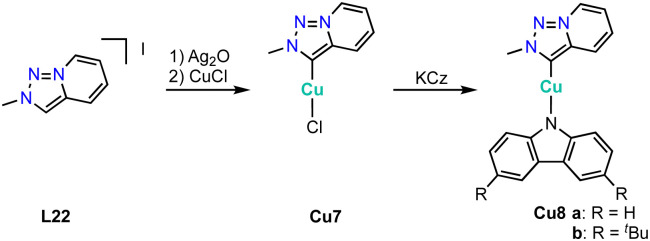
Synthesis of a copper trz complexes Cu8a,b with a carbazole ancillary ligand.

The ancillary ligand in copper(i) trz complexes is amenable to substitutions. Treatment of complex Cu7, prepared from the corresponding triazolium salt L22 and Ag_2_O and subsequent transmetalation, with potassium carbazole (Cz) yields complexes Cu8a,b containing a trz and a Cz ligand (Scheme 42).^135^ In the solid state, Cu8a (R = H) self-assembles into head-to tail dimers due to π–π interactions (3.53 Å) between the electron-rich Cz with the electron-deficient triazole ligand. Analogue Cu8b containing a *t*Bu-substituted Cz ligand displays a similar π–π interaction of 3.39 Å, yet self-assembles into a 1D chain structure instead. A monomer–dimer equilibrium is also manifested in solution through UV-vis absorption, photoluminescence, and 2D NMR data, such as π–π* and n–π* transitions between the Cz and the triazole ligand around 300 nm. Moreover, photoluminescence spectra show two emission bands at 450 and 600 nm. The emission at short wavelength is due to a Cz-based π–π* transition, while concentration-dependent spectra attribute the 600 nm feature to the dimeric structure. This dimer emission band gradually increases in intensity between 273–323 K, and decreases again at higher temperature, as stronger thermal motions disfavor the dimer assembly. Transient photoluminescence studies in THF reveal a dual decay for the emission band of complex Cu8a at 600 nm, comprised of a fast 4.4 ns process and second one at 68.7 ns. In the presence of oxygen, the slow decay is accelerated to 18.4 ns, indicating a decay through a triplet excited state. These data suggest that the fast decay is related to prompt fluorescence of the dimer. As the dimer has a low energy difference between singlet and triplet excited states, efficient (reverse) intersystem crossing occurs and delays the fluorescence decay. The slower decay therefore corresponds to thermally activated delayed fluorescence (TADF).

## Catalytic trends

From the previous sections, some trends for catalytic applications emerge.

**Fig. 5:**
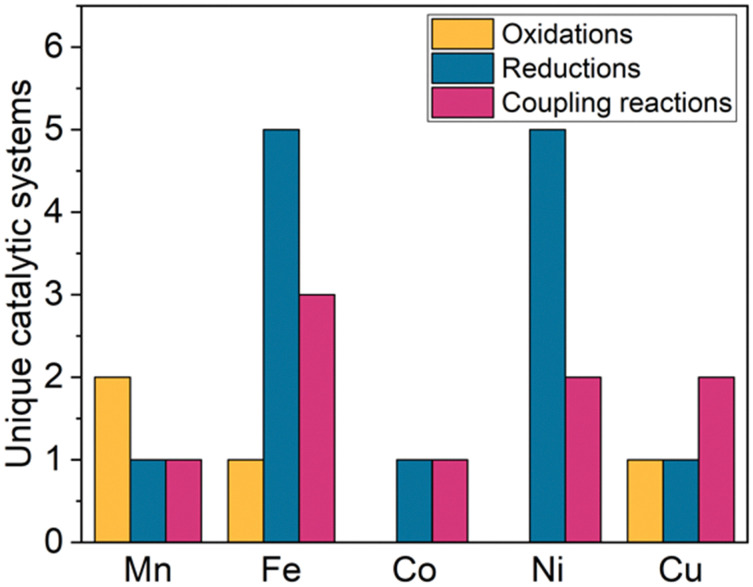
Overview of the various types of catalytic applications of base metals with trz ligands.

(1) Early first-row transition metals with trz ligands are still scarce and applications in catalysis have not been reported thus far. This is remarkable when considering the catalytic competence of these metals, for example in olefin polymerization (group 3 and 4 metals) and in oxidative transformations (group 5), and one might expect progress in these areas in the near future.

(2) With the emergence of manganese as a catalytically active metal in the last decade, also first trz Mn complexes are shown to be useful catalysts, including low-valent manganese complexes for reductive transformations and high-valent systems for oxidations.

(3) Late first-row transition metal trz complexes including Fe, Co, Ni, and Cu have been demonstrated to be versatile catalysts for a diverse range of transformations (Fig. 5)

(4) Despite the robustness of trz ligands and carbenes in general towards oxidations, the largest number of reactions catalyzed by base metal trz complexes are in the broad classes of coupling reactions, and especially reductive transformations, while oxidations are generally still rare and so far absent for nickel and cobalt trz complexes, which provides ample opportunities for further research.

A more specific analysis of reactions catalyzed by first-row metal trz complexes reveals that the most popular reactions include hydrosilylations and CO_2_ conversions (Table 1). For several reactions, metal trz complexes have set the current benchmark in terms of performance and outperform any other homogeneous catalyst. For example, Fe8 is the most active catalyst developd so far for the intramolecular C–H amination using alkylazides. The complex reaches TONs of an order of magnitude higher than any other catalyst.^96^ Likewise, the hydrosilylation of carbonyl substrates with PMHS as a waste-recycled silane approaches 50 000 TON with Fe10 as catalyst precursor, which is unprecedented for first row transition metals and competes favorably with the activity of catalysts based on noble metals.^97^ Other systems are amongst the most active systems in their field, such as the porphyrine-inspired complex Fe7 for epoxidation,^92^ and complex Ni10 with high faradaic efficiency for the electrocatalytic CO_2_ reduction and high selectivity towards formate formation.^119^ This selectivity is highly unusual for first-row transition metals, as they generally produce CO, and holds promise for energy-relevant electrocatalytic hydrogen storage from water.

**Table 1:** Overview of the catalytic reactions performed by first-row transition metal complexes bearing a trz ligand

Catalytic reaction	Catalyst	Conditions	Performance^a^		Ref.
Reductions					
CO_2_ reduction	Mn16	Electrocatalytic −1.94 V	90% FE	170 h^−1^ TOF	81
	Fe2	Piperidine coupling	96 TON	74 h^−1^ TOF	88
	Ni10	Electrocatalytic −1.8 V	83% FE	*k*_obs_ = 370 s^−1^	119
	Ni11	Epoxide coupling	1000 TON	n.d.	121

Hydrosilylation	Fe2	PhSiH_3_, aldehydes, 60 °C	1000 TON	14 400 h^−1^ TOF	40 and 84
	Fe13	(EtO)_3_SiH, aldehydes, 40 °C	20 TON	n.d.	104
	Fe10	PMHS, ketones, 25 °C	36 000 TON	140 h^−1^ TOF	97
	Co7	PhSiH_3_, aldehydes, 40 °C	400 TON	180 h^−1^ TOF	115
	Ni3	PhSiH_3_, aldehydes, 40 °C	6000 TON	23 000 h^−1^ TOF	117
	Ni9	PhSiH_3_, aldehydes, 60 °C	7400 TON	27 000 h^−1^ TOF	120
Hydroboration	Cu6	Cinnamic esters, LiO*t*Bu, 0 °C	10 TON	ee = 60%	134
Alkyne semihydrogenation	Fe2	Me(EtO)_2_SiH, 60 °C	12 TON	n.d.	89
Hydrogen evolution	Ni9	Electrocatalytic, AcOH		*k*_obs_ = 440 s^−1^	119

Oxidations					
Epoxidation	Fe7	H_2_O_2_ and Sc(OTf)_3_	180 TON	41 000 h^−1^ TOF	92
Alkane oxidation	Cu1	H_2_O_2_, 80 °C	40 TON	n.d.	133
Alcohol oxidation	Mn1	TBHP, 40 °C	100 TON	72 h^−1^ TOF	78
	Mn12	TBHP, 60 °C	400 TON	540 h^−1^ TOF	83

Coupling reactions					
C–C cross coupling	Ni1	Suzuki, PhB(OH)_2_, K_3_PO_4_, 110 °C	44 TON	250 h^−1^ TOF	116
	Ni12	Suzuki, PhB(OH)_2_, K_3_PO_4_, 80 °C	15 TON	n.d.	122
Alkyne dimerization	Fe5	Alkyne, LiHMDS	30 TON	n.d.	90
Alkyne coupling/click	Cu4	Halo-click, RT	40 TON	n.d.	131
	Cu5	Click, RT	560 TON	n.d.	132
C–H amination	Fe8	Alkylazides, 120 °C	7600 TON	150 h^−1^ TOF	96
*N*-Alkylation of amines	Mn3	BnOH, KO*t*Bu, 100 °C	67 TON	n.d.	79
Lactide polymerization	Fe2	Neat, 150 °C	4100 TON	*k*_app_ = 0.0085 s^−1^	86
Biginelli condensation	Co7	Benzaldehydes, 100 °C	200 TON	200 h^−1^ TOF	115

a n.d. = not determined.

For many other reactions, the catalytic activities are clearly still at early stages, *e.g.* oxidative functionalizations, classic C–C cross-coupling, or hydroelementation reactions of unsaturated bonds beyond hydrosilylation. These gaps provide challenges and exciting opportunities for further work alike, and they will require consideration of ligands such as trz for imparting catalytic activity and simultaneously also for tailoring reliable coordination to first-row transition metals.

## Conclusions

This overview demonstrates the substantial advances that have been made in recent years both in synthesis and in the catalytic and photochemical application of first-row transition metal complexes upon incorporating trz ligands. While trz complexes of early first-row transition metals is still scarce, mostly because of synthetic challenges and the unfavorable thermodynamics of the M–C bond with these metals, strategies have emerged to form stable complexes based on multidentate trz ligands featuring anionic donor sites as anchors to support the metal carbene bond. In addition, and particularly relevant for mid transition metals such as Cr, Mn, and Fe, the incorporation of strongly π-accepting spectator ligands allows for preparing trz metal complexes. Late first-row transition metals with their softer nature do not require any additional stabilization tactics for the trz to coordinate, though they remain prone to trz protonation. In contrast to early first-row transition metals, for which only coordination chemistry and photochemical properties have been explored thus far, late first-row transition metals show extraordinary activity in various catalytic transformations including: C–H amination, hydrogenation, acetylene dimerization, polymerization, hydrosilylation, CO_2_ reduction and coupling, several C–H oxidations Biginelli reaction and the click reaction. Some of these catalysts are amongst the highest performing systems known to date and showcase the high potential of trz ligands to enable catalytic activity. Various areas are still in their infancy, such as the catalytic application of early transition metal trz complexes, or the enantioselective transformations, areas that may grow rapidly in the future when considering the ease and versatility of trz ligand synthesis.

## Conflicts of interest

There are no conflicts to declare.
